# Short-Term Omega-3 Supplementation Modulates Novel Neurovascular and Fatty Acid Metabolic Proteome Changes in the Retina and Ophthalmic Artery of Mice with Targeted *Cyp2c44* Gene Deletion

**DOI:** 10.3390/cells11213494

**Published:** 2022-11-04

**Authors:** Natarajan Perumal, Anna Herfurth, Norbert Pfeiffer, Caroline Manicam

**Affiliations:** Department of Ophthalmology, University Medical Centre of the Johannes Gutenberg University Mainz, Langenbeckstr. 1, 55131 Mainz, Germany

**Keywords:** Cyp2c44, cytochrome P450, omega-3 fatty acid, ophthalmic artery, proteomics, retina

## Abstract

Cytochrome P450 (CYP) gene mutations are a common predisposition associated with glaucoma. Although the molecular mechanisms are largely unknown, omega-3 polyunsaturated fatty acids (ω-3 PUFA) and their CYP-derived bioactive mediators play crucial roles in the ocular system. Here, we elucidated the proteome and cell-signalling alterations attributed to the main human *CYP2C* gene deficiency using a homologous murine model (*Cyp2c44^−/−^*), and unravelled the effects of acute ω-3 PUFA supplementation in two ocular vascular beds comprising the retrobulbar ophthalmic artery (OA) and retina (R). Male *Cyp2c44^−/−^* mice (KO) and their floxed littermates (WT) were gavaged daily for 7 days with 0.01 mL/g of ω-3 PUFA composed of menhaden fish oil. Another group in respective strains served as vehicle-treated controls. OA and R were isolated at day 8 post-treatment (*n* = 9/group) and subjected to mass spectrometry (MS)-based proteomics and *in silico* bioinformatics analyses. *Cyp2c44^−/−^* resulted in significant detrimental proteome changes associated with compromised vascular integrity and degeneration in the OA and R, respectively. However, notable changes in the OA after ω-3 PUFA intake were associated with the maintenance of intercellular junctional and endothelial cell functions, as well as activation of the fatty acid metabolic pathway in the KO mice. Conversely, ω-3 PUFA supplementation profoundly influenced the regulation of a large majority of retinal proteins involved in the preservation of neuronal and phototransduction activities in WT mice, namely synaptophysin, phosducin and guanylate cyclase-1, while significantly abrogating degenerative processes in the KO mice *via* the regulation of, namely, synaptotagmin-1 and beta-crystallin B2. In gist, this study demonstrated that dietary supplementation with ω-3 PUFA for a short period of seven days regulated specific neuro-vasculoprotective mechanisms to preserve the functionality of the OA and R in the absence of Cyp2c44. The potential adjunct use of ω-3 PUFA for glaucoma therapy needs further investigation.

## 1. Introduction

Dietary supplementation with exogenous metabolites and bioactive compounds of botanical and marine origin has long been a subject of much interest, as well as controversial debate, owing to their profound effects on various physiological mechanisms. In light of these, the long-chain omega-3 polyunsaturated fatty acids (ω-3 PUFA) from natural fish oil have emerged as an essential micronutrient supplement with a myriad of preventative and palliative effects on different disease conditions, including various insidious ocular pathologies, e.g., diabetic retinopathy, age-related macular degeneration (AMD) and glaucoma [1,2,3,4]. The primary ω-3 PUFAs are composed of docosahexaenoic acid (DHA) and eicosapentaenoic acid (EPA). Intriguingly, the former lipid mediator accounts for approximately 20% of the weight of the retina, and unsurprisingly, it is also the main ω-3 PUFA found in photoreceptor cells [5,6,7].

The pleiotropic beneficial effects in the eye exerted by the habitual intake of ω-3 PUFA are reflected in the experimental animal models of ocular diseases, as well as in preclinical studies in population-based cohorts. Although there are a limited number of studies on the applicability of oral supplementation with ω-3 PUFA in glaucoma patients, the attempts to delineate its beneficial effects against retinal dysfunction have been elegantly demonstrated by several groups, showing that these fatty acids do indeed confer protective effects against elevated intra-ocular pressure (IOP)-induced glaucomatous damage in the retina [4,8,9,10,11]. Similarly, other studies have provided compelling experimental evidence supporting the therapeutic efficacy of ω-3 PUFA in ischemic optic neuropathy and AMD [2,12,13,14,15,16]. On the other hand, population-based studies such as the Blue Mountains Eye Study conducted in elderly Australians [17], the Rotterdam Study [18], the French Nutritional AMD Treatment, phase 1 (NAT-1) [19] and the 6-year PREDIMED (Prevención con Dieta Mediterránea) trial [20] have all demonstrated that these essential ω-3 fatty acids appear to be beneficial and can be considered as a potential prophylactic therapeutic regimen for patients with sight-threatening AMD and diabetic retinopathy.

Mechanistically, both DHA and EPA are substrates for the enzymes cyclooxygenase (COX), lipoxygenase (LOX) and cytochrome P450 (CYP) [21,22,23]. However, the CYP isoforms, particularly of the CYP2C subfamily, possess the capacity to preferentially metabolize these ω-3 PUFAs as relatively efficient substrates compared to the other two branches in the cascade [21]. It is well-recognized that the *CYP1B1* gene mutation is one of the driving factors that predisposes to the development of various types of glaucoma in humans, and, although the lack of this gene expression in murine models demonstrated no overt glaucomatous pathology, there was an increased vulnerability of the retinal axons to the disease pathogenesis [24]. The role of the CYP isoforms as well as the lipid metabolites of PUFAs generated by these enzymes in the retina has been studied extensively, with particular focus on the primary human epoxygenases, CYP2C8 and CYP2C9 [25,26,27]. A study by Shao et al. showed that the overexpression of human CYP2C8 in a mouse model of oxygen-induced retinopathy promoted retinal angiogenesis in the group fed with a ω-3 PUFA-enriched diet [27]. The main murine isoform, Cyp2c44, shares a high homology with the human CYP2C8/9, and hence, genetic manipulation of this isoform has been instrumental in unravelling many functional and mechanistic roles of this enzyme in the retina using mouse models. An investigation by Hu and colleagues showed that Cyp2c44 is highly expressed in the retinal Müller glial cells, and endothelial cell proliferation and vessel diameter expansion were promoted in the gene knockout (*Cyp2c44*^−/−^) mice associated with the accumulation of DHA-derived hydroxyl-docosahexaenoic acid (HDHA) in the retina [26]. In retrospect, in an endeavour to further explore the potential role and expression of this CYP isoform in the ocular vasculature, a recent study of ours has provided the first and novel insight that Cyp2c44 is expressed predominantly in the vascular smooth muscle cells of the ophthalmic artery, and the ω-6 arachidonic acid-derived secondary metabolites, consisting of epoxyeicosatrienoic acids (EETs), mediate va-soconstriction [28].

Although many studies were undertaken to decipher the effects of ω-3 PUFAs in various organs, either in animal models or in preclinical human trials, a proteome-wide mapping of the precise changes attributed to the chronic lack of the PUFA-metabolizing CYP enzyme in different ocular vascular beds remains largely unexplored. Therefore, considering the pivotal (patho)physiological roles of the CYP-derived lipid mediators in o-cular blood vessels based on our previous studies, the current study aimed to investigate the molecular mechanisms underlying the beneficial action of oral supplementation with ω-3 PUFAs, consisting mainly of EPA and DHA, in two different ocular vascular tissues comprising the retrobulbar ophthalmic artery and neural retina. We hypothesized that the effects of the *Cyp2c44* gene knockout and ω-3 PUFA are manifested more significantly in the retina compared to the ophthalmic artery. The main aim of the present study was two-fold; first, to explore the protein changes related to a short-term (7 days) oral supplementation with ω-3 PUFA in *Cyp2c44*^−/−^ mice compared to their floxed littermate control, and second, to map the expression of differentially regulated proteins and decipher the cell-signalling pathways in a non-targeted manner to compare the mo-dulations of the retinal and ophthalmic arterial proteome following supplementation.

## 2. Materials and Methods

### 2.1. Animals

This study was approved by the Animal Care Committee of Rhineland-Palatinate (Landesuntersuchungsamt Rheinland-Pfalz, Koblenz, Germany; permission no: 23 177-07/G 20-1-091) and institutional animal care committee [Translational Animal Research Centre (TARC)] of the University Medical Centre of the Johannes Gutenberg University Mainz. Animal experiments were conducted in strict adherence to the Association for Research in Vision and Ophthalmology (ARVO) Statement for the Use of Animals in Ophthalmic and Vision Research and followed the recommendations in the Animal Research: Reporting of In Vivo Experiments (ARRIVE) guidelines. Animal care conformed to institutional guidelines and the EU Directive 2010/63/EU. Animal use in this study was in accordance with the 3R principle.

Floxed Cyp2c44 mice were generated by TaconicArtemis (Cologne, Germany) using C57BL/6 embryonic stem cells for gene targeting, as described previously [28]. The mice were crossed with Gt(ROSA)26Sortm16(Cre)Arte mice expressing Cre under the control of the endogenous Gt(ROSA)26Sor promoter (TaconicArtemis) to generate mice globally lacking Cyp2c44 (*Cyp2c44^−/^^−^*). Breeding pairs were provided by Prof. Ingrid Fleming from the Institute for Vascular Signalling, Goethe University, Frankfurt. The mice were bred and housed in standard autoclaved polypropylene cages with access to mouse chow and water ad libitum under standardised conditions (12 h light/dark cycle, temperature 23 ± 2 °C and relative humidity 55 ± 10%) at the TARC.

### 2.2. Experimental Design

**Figure 1:**
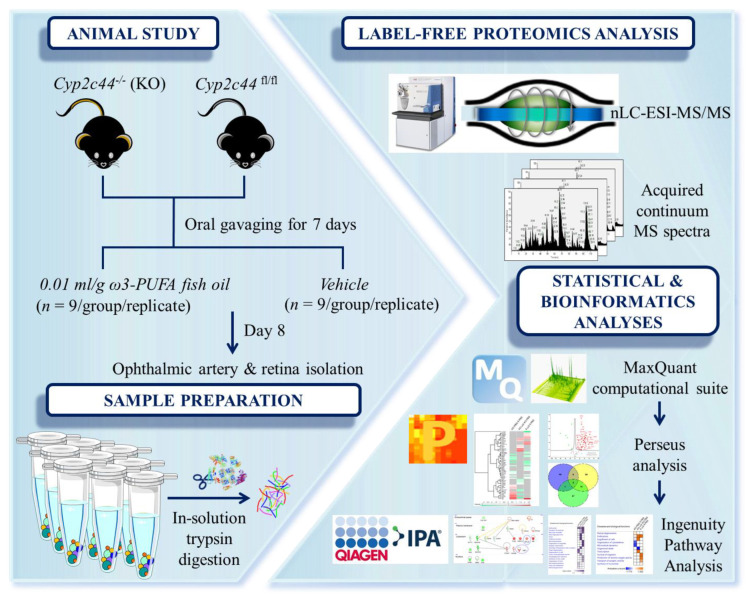
**Workflow overview.** The schematic overview of the experimental design of this study. Ophthalmic artery and retina were harvested from *Cyp2c44^−/−^* (KO) and *Cyp2c44^fl/f^* (floxed littermate control) mice following oral gavaging with either ω-3 PUFA-enriched fish oil or vehicle control for 7 days. Samples were subjected to in-solution trypsin digestion prior to subjecting to bottom-up proteomics profiling employing the nano-LC-ESI-MS/MS. The emerging continuum MS datasets were subjected to robust statistical and in silico bioinformatics analyses, which enabled in-depth data mining to identify differentially expressed protein signatures and, subsequently, elucidate the underlying mechanisms.

The overview of the experimental workflow is represented as a scheme (Figure 1). Briefly, male Cyp2c44^−/−^ (KO) and Cyp2c44^fl/fl^ mice (littermate control) aged 3 to 5 months were used in this study. Mice in the treated group (*n* = 9/group) were orally gavaged once daily for 7 days with 10 µL/g of body weight ω-3 PUFA consisting of menhaden fish oil (F8020, Sigma-Aldrich, St. Louis, MO, USA) and phosphate-buffered saline (PBS, serving as control; *n* = 9/group). The ω-3 PUFA contains 20–31% octadecatrienoic, eicosapentaenoic (EPA) and docosahexaenoic acids (DHA). Animals in the experiment were monitored daily by the experimenters and animal caretakers to ensure their welfare. This included monitoring their body weight and, water and food intake. Daily body weight checks were performed starting from the day before the first oral gavage (day 0) until the day of sacrifice and sampling (day 8).

### 2.3. Sample Preparation

Murine ocular vascular tissue sample preparation was carried out as described elsewhere [29,30]. Briefly, the mice were euthanized with CO_2_ and their eyes were immediately enucleated with optic nerve and extraocular tissues in ice-cold Krebs–Henseleit buffer. The ophthalmic artery and neural retina were carefully isolated and cleaned of surrounding orbital tissues and fat under a stereomicroscope using fine tip-precision tweezers and Vannas capsulotomy scissors. The isolated samples were immediately snap-frozen in liquid nitrogen and stored in −80 °C until use.

### 2.4. Tissue Protein Extraction

The protein-extraction procedure for both the ophthalmic artery and retinal samples was carried out by employing our in-house protocol, which was established specifically for ocular blood vessels [30,31]. Ophthalmic artery samples were pooled from both eyes of three mice per replicate (*n* = 9 mice/group) to yield a sufficient amount of protein for analysis, while the retinal tissues were analysed individually (*n* = 9/group). The samples were homogenized in the T-PER Tissue Protein Extraction Reagent (Thermo Scientific Inc., Waltham, MA, USA) and stainless-steel beads using a Bullet Blender homogenizer (BBY24M Bullet Blender Storm, Next Advance Inc., Averill Park, NY, USA). The supernatant of the tissue homogenates was subjected to buffer exchange and sample cleaning using 3 kDa centrifugal cut-off filters (Amicon Ultra 0.5 mL, Merck Millipore, Carrigtwohill, Ireland). Protein concentration estimation was determined by employing the bicinchoninic acid (BCA) protein assay kit (Pierce, Rockford, IL, USA).

### 2.5. Nano-Liquid Chromatography–Electrospray Ionization–MS/MS (nLC-ESI-MS/MS) Analysis

The nano-LC system employed consisted of an EASY-nLC 1200 system (Thermo Scientific, Rockford, IL, USA) with an Acclaim PepMap RSLC, 75 µm × 50 cm, nanoViper analytical column (Thermo Scientific, Rockford, IL, USA). Solvent A consisted of LC-MS grade water with 0.1% (*v*/*v*) formic acid, and solvent B consisted of LC-MS grade acetonitrile with 20% (*v*/*v*) water and 0.1% (*v*/*v*) formic acid. The run of the resulting gradient per sample added up to a total time of 90 min (0–60 min: 5–30% B, 60–70 min: 30–100% B, 70–90 min: 100% B). The nano-LC system was directly coupled to the ESI-LTQ-Orbitrap-XL-MS system (Thermo Scientific, Bremen, Germany) and continuum mass spectra data were acquired on the ESI-LTQ-Orbitrap-XL MS. The general mass spectrometric conditions were as follows: positive-ion electrospray ionization mode, spray voltage set to 2.15 kV, heated capillary temperature set at 220 °C. The system was used in the data-dependent mode of acquisition, enabling automatic switches between MS and MS/MS modes. In MS mode, the lock mass option was enabled. Internal recalibration was acquired in real time *via* polydimethylcyclosiloxane (PCM) ions (*m*/*z* 445.120025). The LTQ-Orbitrap was operated in a data-dependent mode of acquisition to automatically switch between Orbitrap-MS and LTQ-MS/MS acquisition. Survey full-scan MS spectra (from *m/z* 300 to 2000) were acquired in the Orbitrap with a resolution of 60,000 at *m*/*z* 400 and a target automatic gain control (AGC) setting of 1.0 × 10^6^ ions. The seven most intense precursor ions were sequentially isolated for fragmentation in the LTQ with a collision-induced dissociation (CID) fragmentation; the normalized collision energy (NCE) was set to 35% with an activation time of 30 ms with a repeat count of 2 and dynamic exclusion duration of 210 s. The resulting fragment ions were recorded in the LTQ.

### 2.6. Label-Free Quantification (LFQ) Analysis

The acquired continuum MS spectra were analysed by MaxQuant computational suite (version 2.0.1.0) and its built-in Andromeda search engine for peptide and protein identification [32,33,34]. Tandem MS spectra were searched against the SwissProt database, as follows: *Homo sapiens* (9606), date: 21 May 2021, 20,395 annotated proteins; *Mus musculus* (10090), date: 21 May 2021, 17,073 annotated proteins. Both human and mouse databases were used to maximize protein identification due to the limited availability of annotated proteins in the mouse-specific database [30]. Standard settings with a peptide mass tolerance of ±30 ppm and a fragment mass tolerance of ±0.5 Da, with ≥6 amino acid residues and only “unique peptides” that belong to a protein were chosen [32,34]. Target-decoy-based FDR for peptide and protein identification was set to 0.01 to limit a certain number of peak matches by chance. Carbamidomethylation of cysteine was set as a fixed modification, while protein N-terminal acetylation and oxidation of methionine were defined as variable modifications; enzyme—trypsin and maximum number of missed cleavages—2. The summary of MaxQuant parameters employed in the current analyses for both ophthalmic artery and retina is as tabulated (Supplementary Table S1).

### 2.7. Statistical and Bioinformatics Analysis

The output of the generated “proteingroups.txt” data from the MaxQuant analysis was used for the subsequent statistical analysis with the Perseus software (version1.6.14.0) following data cleaning from reverse hits and contaminants. The statistical analysis in Perseus was performed as follows: First, LFQ intensities were log_2_-transformed and the results were filtered to include only peptides with 100 % valid measured values in at least one of the study groups. Next, missing values were imputated from a normal distribution in standard settings (width: 0.2, down shift: 1.8), enabling statistical analysis [34]. Significantly differentially expressed proteins were identified by a Student’s two-sample t-test with *p* < 0.05. An unsupervised hierarchical clustering analysis was performed according to Euclidean distance (linkage = average; pre-process with k-means) to generate a heat map of the differentially expressed proteins. The gene names of the significantly differentially expressed proteins in each group were used for functional annotation and pathways analyses employing the Ingenuity Pathway Analysis software (v01–04, IPA; Ingenuity QIAGEN Redwood City, CA, USA) [35]. The IPA analysis unravelled the protein–protein interaction (PPI) networks that were experimentally observed and had direct interactions, as well as annotated the proteins according to their subcellular localization and identified the significantly affected canonical pathways and top biological functions associated with the differentially expressed ophthalmic arterial and retinal proteins. The top enriched canonical pathways and biological functions of the differentially expressed proteins were presented with a *p*-value calculated using multiple testing correction, and the significance threshold was set at −log (*p*-value) > 1.3. The IPA Upstream Regulator Analytic tool was used to identify the gamut of molecules predicted to function as upstream regulators (i.e., transcription factors, ligand-dependent nuclear receptors, growth factors, kinases, etc.) and elucidate the expected causal effects between upstream regulators and targeted protein expression changes observed in experimental datasets. The overlap *p*-value measures whether there is a statistically significant overlap between the dataset and the proteins that are regulated by a regulator. It is calculated using Fisher’s Exact Test and the significance threshold is attributed to *p*-values < 0.01. The activation z-score was used to infer the likely activation states (activation or inhibition) of upstream regulators based on comparison with a model that assigns random regulation directions.

## 3. Results

### 3.1. General Observation and ω-3 PUFA-Related Body Weight Changes

Oral gavaging with either ω-3 PUFA or PBS for 7 days did not produce any demonstrable changes or abnormalities in the general appearance and behaviour of the animals. There was no treatment-related mortality or morbidity observed throughout the study period. All mice survived until the day of scheduled sacrifice. The mice were weighed immediately before euthanasia on day 8. Although a slight but non-significant (*p* > 0.05) decrement of body weight was observed in all groups on day 8 compared to the day before the start of oral gavage (day 0), the mean body weight did not differ between groups (KO-ω-3 PUFA: 33.15 ± 1.66 g *cf.* 31.03 ± 1.05 g; KO-PBS: 35.14 ± 2.22 g *cf.* 32.94 ± 1.93 g; floxed-ω-3 PUFA: 30.64 ± 1.29 g *cf.* 27.53 ± 1.43 g; and floxed-PBS: 32.54 ± 1.18 g *cf.* 31.71 ± 0.90 g at day 0 and day 8, respectively) **(**Supplementary Figure S1).

### 3.2. Label-Free Quantitative Proteomics Analysis

Bottom-up LC-MS/MS proteomics analysis assessed the proteome-wide changes attributed to short-term supplementation with ω-3 PUFA and identified a total of 652 and 1377 proteins from the ophthalmic artery and retina, respectively, at less than 1% false discovery rate (FDR < 1%) (complete protein list in Supplementary Tables S2 and S3, respectively). In this study, we employed both mouse and human databases in UniProt for a tandem MS search to maximize protein identification, owing to a limited annotation of murine proteins (17,073 proteins) compared to a higher number of annotated proteins in the human database (20,395 proteins) (Supplementary Table S1). The use of a multiple database search has been demonstrated to provide significantly more protein identification, which is not restricted to only those sequences obtained from the organism of interest [30,36,37,38,39].

### 3.3. Influence of ω-3 PUFA Administration on the Ophthalmic Arterial Proteome

**Figure 2:**
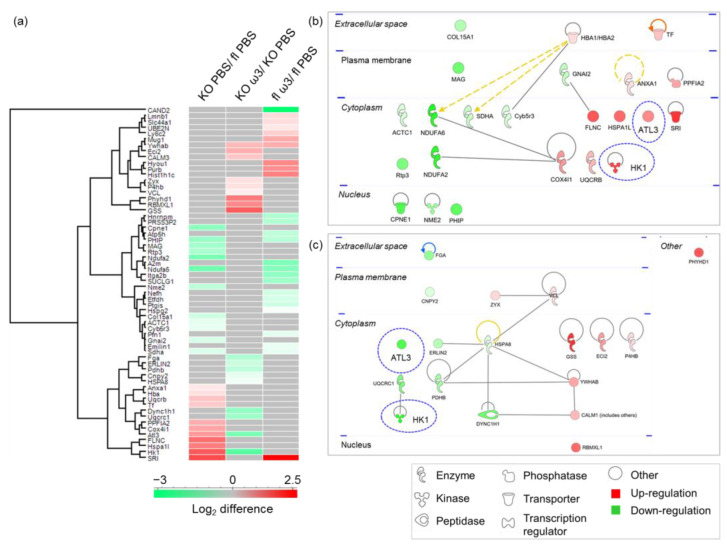
**Differentially expressed protein profiles of the ophthalmic artery.** (**a**) Hierarchical clustering of the differentially expressed proteins represented in a heat map. The up-regulated proteins are shown in red, and the down-regulated proteins are in green. The protein–protein interaction depicts the networks of differentially expressed proteins in the ophthalmic artery of the KO mice (**b**) before and (**c**) after 7-day intake of ω-3 PUFA. Colours red and green represent increment and decrement of protein abundance, respectively, with different colour intensities that correspond to the degree of differential expression. Proteins are annotated according to their localization in different cellular compartments and are depicted as different shapes representing the functional classes of the proteins (e.g., enzyme, transcription regulator, transporter, etc.). Blue dotted circles highlight the distinct change in protein regulation profiles following ω-3 PUFA supplementation.

The vascular proteome changes in the murine ophthalmic artery following acute oral supplementation with ω-3 PUFA were demonstrated by the differential expression of a total of 60 proteins (complete protein list in Supplementary Table S4). As many as 23, 18 and 26 proteins were significantly differentially expressed in the KO PBS/fl PBS, KO ω3/KO PBS and fl ω3/fl PBS group, respectively (Figure 2a). Among the 23 proteins differentially regulated in the KO PBS/fl PBS group, an almost similar number of proteins were up- (11 proteins) and down-regulated (12 proteins), while an equal number of nine proteins were up- and down-regulated in the KO ω3/KO PBS group. In the fl ω3/fl PBS group, a higher number of proteins were down-regulated (16 proteins) compared to 10 up-regulated proteins. The administration of ω-3 PUFA compared to vehicle control resulted in the expression of 21 and 15 exclusive proteins in the floxed and KO mice, respectively, while as many as 17 proteins were exclusively regulated in the vehicle-treated KO mice compared to their floxed littermates fed the same (Supplementary Figure S2a).

Next, the IPA analysis of differentially expressed protein clusters in the KO mice administered with PBS (KO PBS/fl PBS) and ω-3 PUFA (KO ω3/KO PBS) revealed that the majority of proteins comprised enzymes, which were localized in the cytoplasm (Figure 2b,c, respectively). Similarly, the protein–protein interaction networks of the differentially expressed proteins of floxed mice fed with ω-3 PUFA (fl ω3/fl PBS group) demonstrated a high number of cytoplasmic proteins composed of enzymes (Supplementary Figure S3a). Interestingly, two proteins comprising atlastin-3 (Atl3) and hexokinase-1 (Hk1), which were up-regulated in the KO group fed with only vehicle (KO PBS/fl PBS), were significantly down-regulated following administration with ω-3 PUFA in the KO mice (KO ω3/KO PBS) (highlighted with blue dotted circles, Figure 2b,c, respectively). However, only one similar protein (14-3-3 protein beta/alpha, Ywhab) was found to be highly regulated following ω-3 PUFA in both KO and floxed mice.

**Figure 3:**
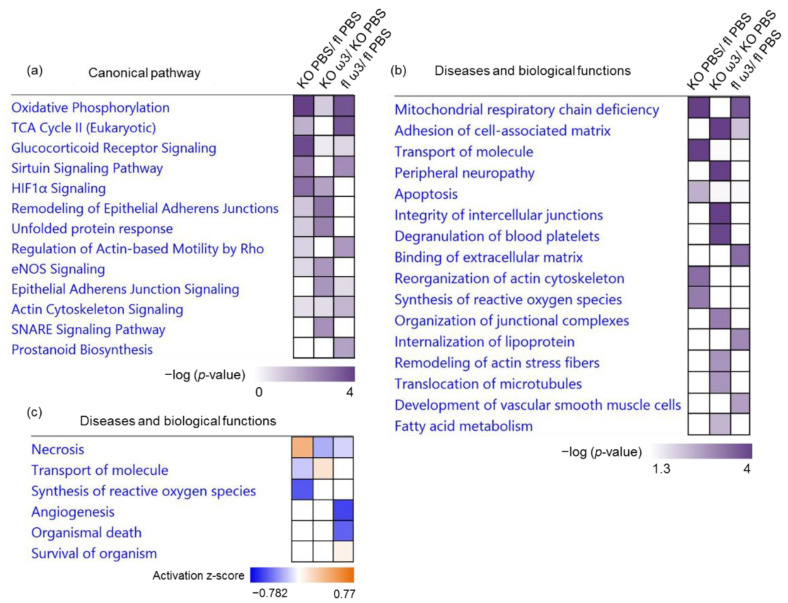
**Top significantly enriched canonical pathways and diseases and biological functions in the ophthalmic artery.** (**a**) The significantly enriched canonical pathways implicated in the ophthalmic artery, which were determined by *p*-value overlap between the proteins identified in our datasets and the molecules in the respective pathways. The significance of enrichment (−log10 (*p*-value), one-sided Fisher’s exact test)) is scaled by colour intensity; *p* < 0.05. The clustering of the most significantly affected diseases and biological functions in the ophthalmic artery depicted according to the (**b**) −log10 (*p-* value) and (**c**) activation z-score. Overall z-scores are represented by the colour orange, which indicates activation, and blue indicates inhibition of the signalling pathways.

Further analysis to unravel the functional relevance of these differentially expressed proteins in the ophthalmic artery demonstrated that the top significantly regulated canonical pathways in the KO mice fed with only vehicle were involved mainly in oxidative phosphorylation (*p* = 5.01 × 10^−8^), glucocorticoid receptor signalling (*p* = 1.58 × 10^−4^), HIF-1α signalling (*p* = 8.91 × 10^−4^), sirtuin signalling pathway (*p* = 2.37 × 10^−3^) and TCA cycle (*p* = 2.11 × 10^−2^). On the other hand, the top five canonical pathways that showed significant alteration as a consequence of diet supplementation with ω-3 PUFA to the KO mice involved the remodelling and signalling of the epithelial adherens junctions (*p* = 1.20 × 10^−3^ and 6.18 × 10^−3^, respectively), unfolded protein response (*p* = 2.08 × 10^−3^), SNARE signalling pathway (*p* = 4.67 × 10^−3^) and endothelial nitric oxide synthase (eNOS) signalling (*p* = 6.33 × 10^−3^). Further analysis to identify the specifically affected canonical pathways that were significantly (*p* < 0.05) altered following treatment with ω-3 PUFA in the floxed mice showed an interesting trend that closely mimicked the KO mice treated with PBS. These comprised the significant involvement of proteins implicated in oxidative phosphorylation (*p* = 2.38 × 10^−4^), TCA cycle (*p* = 2.86 × 10^−4^) and the sirtuin signalling pathway (*p* = 3.86 × 10^−3^). Two pathways that were only exclusively involved in the ω-3 PUFA-treated floxed but not in the KO mice were composed of the regulation of actin-based motility by Rho (*p* = 7.09 × 10^−3^) and prostanoid biosynthesis (*p* = 1.09 × 10^−2^) (Figure 3a).

Correspondingly, the disease and biological functional analysis of the differentially expressed proteins showed that most protein clusters in both PBS-treated KO and ω-3 PUFA-treated floxed mice were significantly associated with mitochondrial respiratory chain deficiency (*p* = 3.77 × 10^−8^ and 2.02 × 10^−4^, respectively). On the other hand, the treatment of ω-3 PUFA resulted in significant regulation proteins involved in adhesion of the cell-associated matrix in both KO and floxed mice (*p* = 7.06 × 10^−5^ and 6.18 × 10^−3^, respectively). Among the biological functions that were found to be only regulated by the administration of ω-3 PUFA to the KO mice, the top functions were implicated in peripheral neuropathy (*p* = 3.02 × 10^−6^), integrity of intercellular junctions (*p* = 2.96 × 10^−5^), degranulation of blood platelets (*p* = 1.22 × 10^−4^), organization of junctional complexes (*p* = 7.57 × 10^−4^), remodelling of actin stress fibres (*p* = 1.51 × 10^−3^) and fatty acid metabolism (*p* = 4.35 × 10^−3^) (Figure 3b). Noteworthy, necrosis was activated (z-score = 0.391) and, molecular transportation and synthesis of reactive oxygen species (ROS) were inhibited in the KO mice (z-score = −0.169 and −0.520, respectively). Remarkably, the administration of ω-3 PUFA resulted in the reversal of the former two processes (necrosis and molecular transportation) in the KO mice (z-score = −0.253 and z-score = 0.147, respectively). The ophthalmic arterial proteins of floxed mice treated with ω-3 PUFA were shown to be involved in the inhibition of several cellular processes, namely necrosis (z-score = −0.136), angiogenesis (z-score = −0.577) and organismal death (z-score = −0.487), while consistently activating the organismal survival (z-score = 0.068) (Figure 3c). The gene heatmap of these disease and functional annotations showed that a myriad of different proteins were responsible for the activation and inhibition of the same function in the different treatment groups. For example, only a small proportion of the differentially expressed proteins (i.e. sorcin (SRI), Ywhab, Hk1 and pleckstrin homology domain-interacting protein (PHIP)) were observed to be similar between the groups in mediating the necrotic process (Supplementary Figure S4a), and in the transport of molecules (Supplementary Figure S4d), while the large majority of protein expression differed between the treatment groups and genotypes. However, distinct clusters of protein were found only in several categories, such as in significant regulation of the fatty acid metabolism and inhibition of organismal death in the ω-3 PUFA-administered KO and floxed mice, respectively (Supplementary Figure S4b,c, respectively).

### 3.4. Effects of ω-3 PUFA Supplementation on the Retinal Proteome

**Figure 4:**
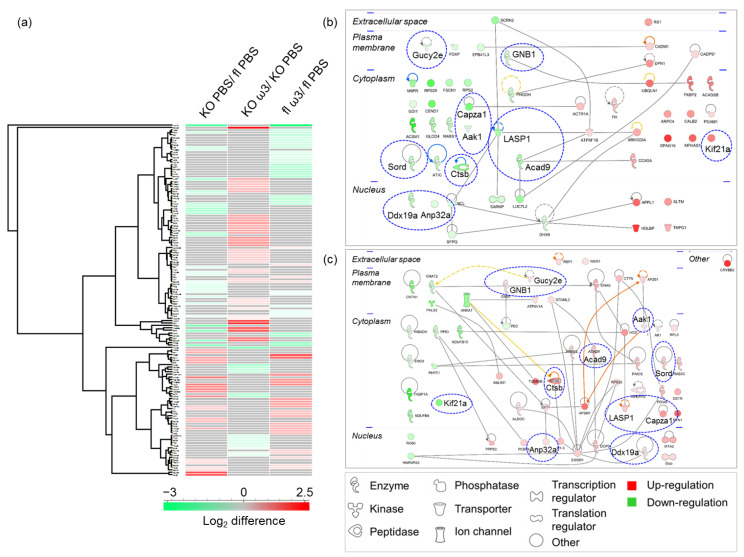
**Profiles of differentially expressed retinal proteins.** (**a**) Heat map depicts the hierarchical clustering of the differentially expressed retinal proteins. Red and green shading indicate up- and down-regulation of the proteins, respectively. The protein–protein interaction networks of differentially expressed proteins in the retina of the KO mice (**b**) before and (**c**) after 7-days of ω-3 PUFA intake. Nodes (proteins) depicted with different shapes represent functional classes of the proteins (e.g., enzyme, transcription regulator, transporter, etc.) and are annotated according to their localization in different cellular compartments. The intensity of the node colour indicates the degree of differential regulation, with the colours red and green representing the increment and decrement of protein abundance, respectively. Blue dotted circles highlight the distinct alteration in protein regulation profiles following ω-3 PUFA supplementation.

Expression profiling identified 144 retinal proteins as significantly differentially expressed (*p* < 0.05) between the different groups of mice supplemented with ω-3 PUFA and vehicle (full list in Supplementary Table S5). Among these, a higher number of proteins (61 proteins) were significantly differentially expressed in the ω-3 PUFA-administered floxed mice compared to the ω-3 PUFA- and vehicle-administered KO mice (56 and 51 proteins, respectively), as represented in the heat map (Figure 4a). An almost similar number of proteins were exclusively differentially expressed in both the ω-3 PUFA-treated floxed and KO group (floxed: 47 proteins and KO: 45 proteins), whereas only 30 proteins were exclusive to the PBS-administered KO mice (Supplementary Figure S2b). Unlike the ophthalmic artery, which lacked any overlapping proteins in all three groups, two retinal proteins were found to be differentially expressed in all groups that consisted of retinal guanylyl cyclase 1 (Gucy2e) and adaptor-associated protein kinase 1 (Aak1). In the vehicle-treated KO and ω-3 PUFA-administered floxed group, the number of down-regulated proteins was higher (29 and 34 proteins, respectively) than the ω-3 PUFA-treated KO mice, with 15 down-regulated and 41 up-regulated proteins (Supplementary Table S5). According to their functional and cellular compartment classification, the majority of these proteins were enzymes localized in the cytoplasm, similar to the ophthalmic artery. However, compared to the ophthalmic artery, a bigger proportion of these proteins (10 proteins), which were significantly down-regulated in the KO mice, were up-regulated in the KO mice following ω-3 PUFA administration. On the contrary, only one up-regulated protein in the former group, kinesin-like protein KIF21A (Kif21a), was down-regulated in the latter (Figure 4b,c). The PPI of the differentially expressed proteins in the ω-3 PUFA-administered floxed group showed that three proteins localized in the nucleus comprising the transcription regulators catenin beta-1 (CTNNB1) and prohibitin (Phb), and an enzyme, the Parkinson disease protein 7 homolog (Park7), have the highest number of interactions with other proteins (Supplementary Figure S5).

**Figure 5:**
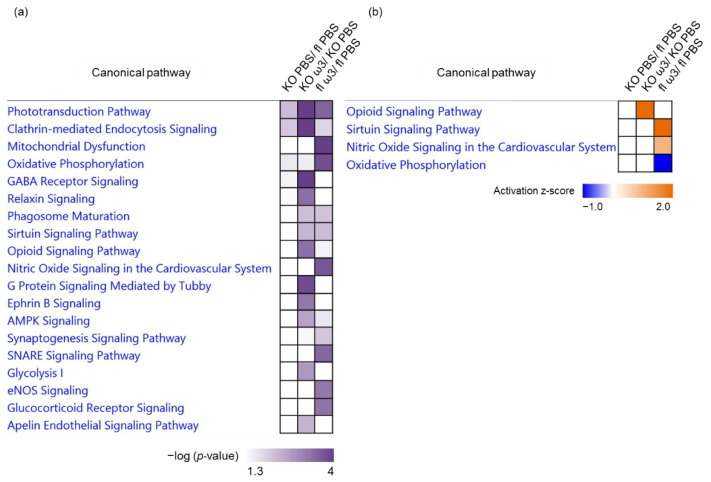
**Top significantly affected canonical pathways in the retina.** Chart depicts the significantly (*p* < 0.05) enriched canonical pathways implicated in the retina, which were determined by *p*-value overlap between the proteins identified in our datasets and the molecules in the respective pathways, according to (**a**) −log10 (*p*-value) and (**b**) activation z-score. Blue indicates negative and orange indicates positive regulation based on the activation z-scores for canonical pathway comparison between the different groups and treatment paradigms.

To further decipher the key mechanisms by which dietary supplementation with ω-3 PUFA alters the proteome of the retina, a functional clustering analysis highlights the significant involvement of several key canonical pathways. The top canonical pathways ranked by *p*-value in the KO mice are depicted by the significant regulation of proteins in the phototransduction pathway (*p* = 6.00 × 10^−3^), clathrin-mediated endocytosis signalling (*p* = 8.22 × 10^−3^) and oxidative phosphorylation (*p* = 2.37 × 10^−2^). It is noteworthy that three pathways comprising glycolysis (*p* = 1.84 × 10^−3^), G-protein signalling mediated by Tubby (*p* = 1.53 × 10^−4^) and GABA receptor signalling (*p* = 1.54 × 10^−5^) were significantly affected only in the KO group, particularly following the administration of ω-3 PUFA (Figure 5a). Remarkably, the involvement of a higher number of canonical pathways was observed in the retina of mice administered with ω-3 PUFA, regardless of the genotype, with an overall trend of activation, as evidenced by the positive z-scores of three major pathways comprising the opioid signalling pathway (*p* = 5.09 × 10^−4^; z-score = 2.0) in the KO group, and the sirtuin (*p* = 5.96 × 10^−3^; z-score = 2.0) and nitric oxide signalling pathways (*p* = 2.19 × 10^−4^; z-score = 1.0) in the floxed group. Only one canonical pathway was shown to have a decreased activity in the ω-3 PUFA-fed floxed group, which wasoxidative phosphorylation (*p* = 1.68 × 10^−4^; z-score = −1) (Figure 5b).

**Figure 6:**
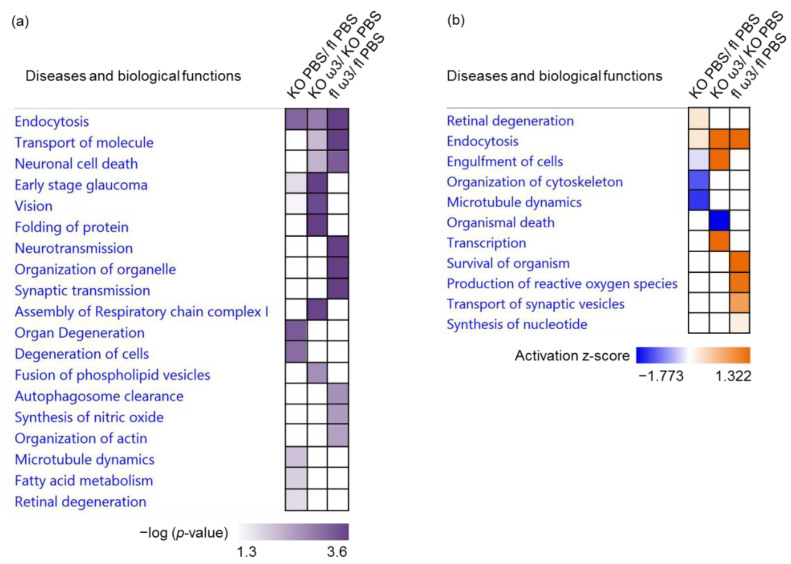
**Top significantly affected diseases and biological functions in the retina.** The clustering of the most significantly affected diseases and biological functions in the retina depicted according to the (**a**) −log10 (*p*-value) and (**b**) activation z-score. Overall z-scores are represented by the colour orange, which indicates activation, and blue indicates inhibition of the signalling pathways.

Subsequent analysis of the disease and biological functions associated the differentially expressed proteins to visual functions and degeneration in the KO mice, namely retinal degeneration (*p* = 1.82 × 10^−2^), cellular and organ degeneration (*p* = 9.05 × 10^−4^ and *p* = 5.63 × 10^−4^, respectively), early-stage glaucoma (*p* = 1.91 × 10^−2^), microtubule dynamics (*p* = 9.46 × 10^−3^) and fatty acid metabolism (*p* = 1.33 × 10^−2^). On the contrary, the significantly affected functions following ω-3 PUFA supplementation in the KO mice were implicated in protein folding (*p* = 1.54 × 10^−5^), the assembly of respiratory chain complex I (*p* = 3.13 × 10^−4^) and the fusion of phospholipid vesicles (*p* = 2.35 × 10^−3^). The differentially expressed proteins in the floxed mice fed with ω-3 PUFA exhibited an interesting pattern of significantly affected functionalities involved in neurotransmission (*p* = 1.95 × 10^−5^), synaptic transmission (*p* = 2.11 × 10^−4^), the organization of organelles and actin (*p* = 2.17 × 10^−5^ and *p* = 3.81 × 10^−3^, respectively), autophagosome clearance (*p* = 2.48 × 10^−3^) and the synthesis of nitric oxide (*p* = 3.25 × 10^−3^) (Figure 6a). A striking feature of these results is that the retinal proteins in the KO mice were involved in the activation of retinal degeneration (z-score = 0.225) and inhibition of pivotal cellular processes, such as organization of the cytoskeleton (z-score = −1.184) and microtubule dynamics (z-score = −1.412). Conversely, the administration of ω-3 PUFA to KO mice resulted in the activation of endocytosis (z-score = 1.287), which was also observed to be highly activated in the floxed mice (z-score = 2.024), the activation of the transcription activities in the retinal cells (z-score = 2.052), and the engulfment of cells (z-score = 1.406), which was inhibited in the vehicle-treated KO mice (z-score = −0.243) (Figure 6b). The gene heatmap depicting the proteins involved in key functions such as significant activation of retinal degeneration in the KO group was attributed to two up-regulated (retinoschisin (Rs1) and ubiquilin-1 (Ubqln1)) and two down-regulated (Gucy2e and GNB1) proteins (Supplementary Figure S6a). The majority of proteins involved in the modulation of the two most affected biological functions, specifically in the ω-3 PUFA-administered KO mice—which were the activation of transcription (Supplementary Figure S6b) and inhibition of organismal death (Supplementary Figure S6c)—were shown to up-regulated. Among these up-regulated proteins, one protein in the former (GNB1) and three in the latter function (cathepsin B, Ctsb; acidic leucine-rich nuclear phosphoprotein 32 family member A, Anp32a and GNB1) were shown to be down-regulated in the KO group, which reflected the germane effects of the ω-3 PUFA treatment in the KO mice. Intriguingly, the protein expression profile of activated organismal survival function in the floxed mice fed with ω-3 PUFA showed that, apart from the exclusive expression of seven proteins, there was also an up-regulation of two proteins common to the KO mice (Rho GDP-dissociation inhibitor 1 (Arhgdia) and epsin-1 (Epn1)) (Supplementary Figure S6d).

### 3.5. The Role of Predicted Upstream Regulators in the Ophthalmic Artery

**Figure 7:**
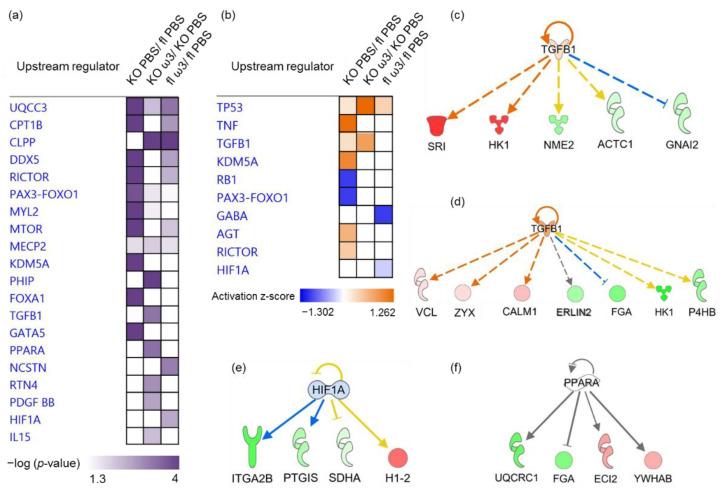
**Predicted upstream regulators in the ophthalmic artery.** The predicted profiles of top selected upstream regulators illustrated according to (**a**) −log10 (*p*-value), *p* < 0.05 and (**b**) activation z-score. Exemplary profiles of significantly affected predicted upstream regulators for the various clusters of differentially expressed proteins represented by (**c**) activated TGF-β1 in the KO group, (**d**) ~3-fold activation of TGF-β1 in the ω-3 PUFA-fed KO group, (**e**) inhibited HIF-1α in the ω-3 PUFA-fed floxed mice and (**f**) significant regulation by PPARα. The colours red and green of the nodes represent the up- and down-regulation of the differentially expressed proteins implicated in the interaction networks, respectively. The predicted activity of the regulators is shown as orange (activated) and blue (inhibited), and grey indicates indeterminable activity pattern.

Finally, the role of upstream molecules, including transcription factors—which were predicted to significantly modulate the downstream expression of specific clusters of differentially expressed proteins—was investigated. In the ophthalmic artery, the common upstream regulators found to be significant in all three groups were UQCC3, MECP2 and PAX3-FOXO1. Among these, the former was also the most significant regulator in the KO group (*p* = 5.68 × 10^−9^), while PHIP was the highly significant and exclusive regulator in the KO group fed with ω-3 PUFA (*p* = 1.26 × 10^−5^). In addition, KDM5A (*p* = 9.43 × 10^−6^), FOXA1 (*p* = 2.31 × 10^−5^ and GATA5 (*p* = 6.54 × 10^−5^) all exclusively governed downstream proteins in the KO group. On the other hand, RTN4 (*p* = 1.29 × 10^−3^), PDGF-BB (*p* = 2.51 × 10^−3^) and IL-15 (*p* = 5.73 × 10^−3^) were all exclusively involved in the regulation of proteins in the ω-3 PUFA-administered KO group. Two upstream regulators exerted exclusive control on the differentially proteins in the ω-3 PUFA-administered floxed group comprising a transcription regulator, Hif-1α (*p* = 2.94 × 10^−3^), and a peptidase, NCSTN (*p* = 7.28 × 10^−4^). In both groups administered with ω-3 PUFA, only one common regulator was identified (CLPP, *p* = 2.79 × 10^−5^ and *p* = 7.74 × 10^−5^ in the KO and floxed mice, respectively) (Figure 7a).

In the KO group, the majority of upstream regulators were activated comprising TP53 or p53 (*p* = 1.23 × 10^−2^; z-score = 0.218), TNF (*p* = 4.72 × 10^−2^; z-score = 1.219), a growth factor TGF-β1 (*p* = 4.63 × 10^−2^; z-score = 0.277), KDM5A (*p* = 9.43 × 10^−6^; z-score = 1.0), AGT (*p* = 4.72 × 10^−2^; z-score = 0.651) and RICTOR (*p* = 9.20 × 10^−6^; z-score = 0.447), whereas only two regulators comprising RB1 (*p* = 1.69 × 10^−3^; z-score = −1) and PAX3-FOXO1 (*p* = 1.81 × 10^−4^; z-score = -1) were inhibited. Both p53 (*p* = 5.24 × 10^−4^; z-score = 1.912) and TGF-β1 (*p* = 5.37 × 10^−4^; z-score = 0.816) were also activated in the KO mice fed with ω-3 PUFA. The former regulator was also found to be activated (*p* = 4.59 × 10^−3^; z-score = 0.378) in the floxed mice fed with ω-3 PUFA, in addition to inhibited GABA (*p* = 1.19 × 10^−3^; z-score = −1) and Hif-1α (*p* = 2.94 × 10^−3^; z-score = −0.254) (Figure 7b).

The exemplary profiles of the proteins governed by activated TGF-β1 in the KO (Figure 7c) and ω-3 PUFA-administered KO group (Figure 7d) showed the downstream regulation of a large cluster of enzymes and proteins with other molecular functions. On the other hand, inhibited transcription regulator Hif-1α was predicted to modulate a cluster of four proteins in floxed mice fed with ω-3 PUFA (Figure 7e), while PPARα was predicted to regulate another four proteins in the ophthalmic artery of ω-3 PUFA-administered KO mice (Figure 7f).

### 3.6. The Role of Predicted Upstream Regulators in the Retina

**Figure 8:**
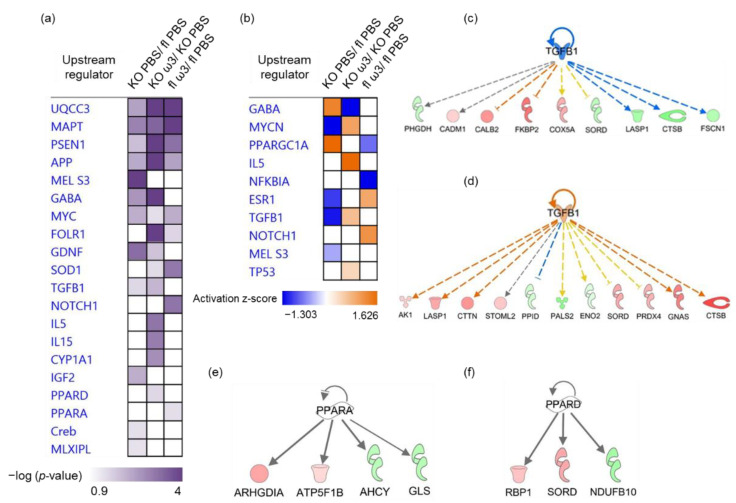
**Predicted upstream regulators in the retina.** The profiles of top selected predicted upstream regulators according to (**a**) −log10 (*p*-value), *p* < 0.05 and (**b**) activation z-score. Exemplary profiles of significantly affected predicted upstream regulators for the various clusters of differentially expressed proteins represented by interaction networks of (**c**) inhibited TGF-β1 in the KO group, (**d**) activated TGF-β1 regulation in ω-3 PUFA-fed KO mice, (**e**) significant regulation by PPARα and (**f**) PPARδ. Red and green colours of the proteins represented by functionality nodes indicate up- and down-regulation of the differentially expressed proteins, respectively. The predicted activity of the regulators is shown as orange (activated) and blue (inhibited), and grey indicates an indeterminable activity pattern.

In comparison to the ophthalmic artery, the involvement of common predicted upstream regulators in all three groups was higher in the retina, as evidenced by the significant modulation by five regulators comprising UQCC3, APP, MAPT, a transcription regulator, MYC and a peptidase, PSEN1. In the KO group, a kinase inhibitor, MEL S3 (*p* = 4.99 × 10^−9^), a growth factor, IGF2 (*p* = 6.37 × 10^−3^), a transcription regulator, MLXIPL (*p* = 4.27 × 10^−2^) and Creb (*p* = 3.93 × 10^−2^) were shown to be exclusive to this group. Likewise, in the ω-3 PUFA-administered KO group, a cluster of cytokines composed of IL-5 (*p* = 7.61 × 10^−4^) and IL-15 (*p* = 1.04 × 10^−3^), an enzyme, CYP1A1 (*p* = 1.98 × 10^−3^) and a ligand-dependent nuclear receptor, PPARδ (*p* = 2.93 × 10^−2^) were predicted to be exclusive in this group. On the contrary, only two regulators comprising a transcription regulator, NOTCH1 (*p* = 7.46 × 10^−4^) and a ligand-dependent nuclear receptor, PPARα (*p* = 3.79 × 10^−2^) were exclusively implicated in the regulation of proteins in the ω-3 PUFA-administered floxed mice (Figure 8a).

The striking effects of short-term ω-3 PUFA-administration in the KO mice were reflected in the reversal of the activity of several upstream regulators. The most prominent results were shown in the inhibition of GABA (z-score = −2.646) and activation of Mycn (z-score = 1) and TGF-β1 (z-score = 0.711) following ω-3 PUFA supplementation. In the floxed mice, the ramification of ω-3 PUFA-administration was reflected in the significant regulation of several transcription factors, particularly the inhibition of PPARGC1A or PGC-1α (*p* = 7.01 × 10^−3^; z-score = −0.742) and NF-κBIα (*p* = 7.60 × 10^−3^; z-score = −2.183), and the activation of NOTCH1 (*p* = 7.46 × 10^−4^; z-score = 1.171) (Figure 8b).

It is intriguing that the proteins predicted to be regulated by the same upstream regulator differed following ω-3 PUFA administration in the same genotype. This was exemplified by the regulation profiles of TGF-β1 in the KO mice without (Figure 8c) and with (Figure 8d) ω-3 PUFA supplementation. It is also noteworthy that two different peroxisome proliferator-activated receptor isoforms were predicted to be involved in the regulation of several differentially expressed retinal proteins following the intake of ω-3 PUFA, exemplified by PPARα in the floxed group (Figure 8e) and PPARδ (Figure 8f) in their KO counterparts.

## 4. Discussion

The current study explored the molecular repercussions and alterations in cell-signalling pathways following a short-term dietary intake of ω-3 PUFA on two major ocular vascular systems comprising the retrobulbar ophthalmic artery and neural retina in an unbiased manner. Additionally, the effects of supplementation in the chronic lack of the main human *CYP2C* gene were investigated using a homologous gene-knockout murine model (*Cyp2c44^−/−^*). To the best of our knowledge, this is the foremost study that investigated in-depth whether ω-3 PUFA supplement elicited acute changes at the proteome level in two different neurovascular beds in the eye within a very short period of 7 days. Although the short-term effects of the intake of ω-3 PUFA supplementation to improve various pathologies, including several ocular diseases, have been a topic of much investigation in the last few decades, these studies were all conducted for a longer period of time compared to our timeline, ranging from a minimum of 2 weeks up to 3 months [2,3,40,41,42,43,44,45]. Since it has been shown that the uptake of radiolabelled DHA into the photoreceptors already begins at 1 h post-ingestion [46], we hypothesized that the profound effects conferred by ω-3 PUFAs in the ocular tissues can be elucidated after 1 week of administration. In the present study, we opted for a ω-3 PUFA source containing a mixture of both EPA and DHA, rather than using EPA or DHA alone, because a combined intake of both ω-3 PUFAs has been shown to be physiologically potent and has an immense beneficial impact, such as a pronounced anti-inflammatory effect [40,41,47]. Combined EPA and DHA supplementation has been demonstrated to protect against retinal damage in a Stargadt disease mouse model [40], AMD [3], myopia [41], age-associated retinal degeneration [2], dry eye disease [45], as well as being recommended for patients with coronary heart disease [48] and depressive symptoms [43].

One of the overt effects of acute supplementation with ω-3 PUFA in the ophthalmic artery of KO mice was manifested in the significant regulation of two proteins, atlastin-3 (Atl3) and hexokinase-1 (Hk1), which were down-regulated following ω-3 PUFA intake. The Atl3 protein belongs to a conserved family of large, dynamin-related GTPases involved in the formation and maintenance of the endoplasmic reticulum (ER) [49,50]. Atlastins, particularly Atl3, are predominantly enriched in the three-way junctions of the ER network and are involved in the biogenesis and regulation of lipid droplets and sterols [49,51]. Lipid droplets are the primary organelles for the storage of fat in cells to accommodate the excess energy in the form of triglycerides and cholesterols within the membrane phospholipid bilayer [52]. An important finding that the structural integrity of the ER is dependent on nitric oxide (NO) in the pulmonary arterial endothelial cells was elucidated by Lee and colleagues [50]. Similar to the source of ω-3 PUFA used in our study and the significant regulation of proteins associated with the eNOS signalling pathway in the ophthalmic artery of KO mice, the administration of menhaden fish oil to Sprague-Dawley rats for 8 weeks demonstrated an up-regulation of the eNOS pathway in the aorta, which was attributed to the maintenance of vascular homeostasis by improving endothelial functions [53]. Among other functions, NO generated by eNOS is also known to increase glycolysis by increasing the activity of glycolytic enzymes such as hexokinases [54]. Hexokinase-1 (HK1) plays an indispensable function as the first rate-limiting housekeeping glycolytic enzyme, which catalyses glucose [55,56]. A significant increment in HK expression was reported following an ischemic episode in the heart, which was presumably attributed to enhanced biosynthetic pathways for regeneration and growth [57]. Similarly, the expression of both HK1 gene and protein were increased in a pulmonary hypertension model in rats [58]. The up-regulation of HK1 promoted glucose metabolism and accelerated the synthesis and accumulation of intracellular fat, which is stored as lipid droplets in the hepatocytes [59]. Another study by Flachsbart et al. reported that the overexpression of HK1 decreased the expression of one of the *CYP2C* genes, which corroborated with our findings in the *Cyp2c44^−/−^* mice [60].

Further dissection of the biological functions of the ophthalmic artery yielded several remarkable changes following administration of ω-3 PUFA, particularly the amelioration of the necrotic process in both mouse genotypes (floxed littermates and KO), as well as the significant activation of molecular transportation that was found to be impeded in the KO mice. Additionally, considering the dynamic role of marine ω-3 PUFA as natural ligands for numerous transcription factors and nuclear receptors [61], we delved further to unravel the roles of potential upstream regulators predicted to orchestrate the downstream regulation of the differentially expressed proteins in the artery. Among the litany of regulators that were exclusively connected to the proteins expressed as a result of ω-3 PUFA administration, the peroxisome proliferator activated receptor-alpha (PPARα) was found to be one of the key players in the KO mice. This is of interest in the current investigation because dietary PUFAs are known to have a direct interaction with this ligand-activated transcription factor, which has crucial metabolic repercussions on fatty acid oxidation [22,62,63] and the regulation of lipid transporters [64]; both reciprocal findings are highlighted in our study as a result of ω-3 PUFA administration in the KO mice. The ablation of PPARα often exacerbated the impairment of fatty acid metabolism, which in turn is compensated by increasing glycolytic products for oxidative phosphorylation (OXPHOS) [62,65]. In keeping with previous studies, the highly significant regulation of OXPHOS in the absence of fatty acid metabolism and PPARα can be attributed to the effects of the *Cyp2c44* gene knockout in the ophthalmic artery in our present study.

On the other hand, fatty acid oxidation is utilized by quiescent endothelial cells as a vasculoprotective metabolic mechanism against oxidative stress [66], and it is also up-regulated during vascular network formation [67]. It is well recognized that vascular endothelial cells play pivotal roles in the maintenance of the vascular barrier and regulation of vasoactive mediators in the ophthalmic artery [28,29,68]. Hence, endothelial cell dysfunction is a major cause of numerous pathologies, including glaucoma [28,29,30,66]. The importance of ω-3 PUFA in the maintenance of endothelial cell function in the ophthalmic artery of KO mice is further demonstrated by the activation of a growth factor with pleiotropic functions, transforming growth factor-beta 1 (TGF-β1). The primary target cell for this multifunctional prototypic member of a highly conserved superfamily is the endothelial cells, as it plays a crucial role in regulating the interplay between vascular endothelial and smooth muscle cells [69,70]. Although TGF-β1 is also activated in Cyp2c44-deficient mice, the magnitude of predicted activation was almost 3-fold in this genotype following the intake of ω-3 PUFA. The complexity of TGF-β1 signalling is reflected in its inherent capacity to elicit diverse context-dependent cellular functions to regulate normal homeostasis [71]. TGF-β1 is also known to be involved in the regulation of endothelial synthesis of vasoactive agents such as NO [72], which was shown to be another canonical pathway significantly regulated in the ω-3 PUFA-fed KO group. As such, albeit the absence of a direct mechanistic link, it is plausible that the activation of TGF-β1 in the KO artery may be one of the acute mechanisms to maintain vascular integrity [73], while ω-3 PUFA augmented its downstream regulation of protein repertoire to retain blood vessel stability [74]. In the floxed mice, despite the nuanced inhibition of the upstream regulator hypoxia-inducible factor-1α (HIF-1α) elicited by ω-3 PUFA, this finding is supported by the inhibition of angiogenesis, with the involvement of a common protein (prostacyclin synthase (PTGIS)) in both signalling pathways. Our inferences were strengthened on the basis of the role of HIF-1α in driving the activation of angiogenic genes [75], as well as the coordinated regulation of PTGIS in endothelial cells, which has a noteworthy function in the modulation of vascular tone [76,77].

Given the preponderance of evidence supporting the effectiveness of ω-3 PUFA in the ophthalmic artery, we further explored its influence on the retinal proteome and found that the effects of ω-3 PUFA supplementation were more profound in the latter than in the former ocular blood vessel, as hypothesized. The first line of results that conservatively illustrated the beneficial effects of ω-3 PUFA in the retina was reflected in the higher number of differentially expressed proteins. Among these, the regulation of several proteins in the KO group noticeably changed after ω-3 PUFA intake, such as retina-specific guanylate cyclase-1 protein (Gucy2e) and Kif21a, while the expression of others was exclusive only upon the ingestion of ω-3 PUFA in both genotypes, namely, phosducin (Pdc), beta-crystallin B2 (Crybb2), synaptophysin (Syp) and synaptotagmin-1 (SYT1). The retina-specific protein Gucy2e, which was down-regulated in the KO group, significantly increased following ω-3 PUFA intake. This gene product of the murine homolog of the human *GUCY2D* is commonly expressed in the outer segment of photoreceptors and plays an indispensable role in phototransduction by replenishing the levels of the secondary messenger, cyclic guanosine monophosphate (cGMP), after light exposure [78,79,80]. Hence, mutations in this gene result in photoreceptor degeneration [81,82]. Another photoreceptor-specific protein, Pdc [83,84], was also found to be differentially expressed only in the ω-3 PUFA-fed floxed and KO groups. Correspondingly, both Gucy2e and Pdc were significantly connected to the phototransduction canonical pathway, and the increment in Gucy2e protein expression was also implicated in mediating the vision function together with Crybb2 in the ω-3 PUFA-fed KO mice, corroborating the findings of Georgiou and Prokopiou, where AMD patients supplemented with EPA and DHA demonstrated a significant improvement of vision [85].

The Crybb2 protein isoform inherently possesses strong neuroprotective and regenerative potential and is usually expressed in regenerating retina [86,87]. Moreover, Crybb2 was found to be co-localized with a Ca^2+^-sensing synaptic vesicular protein, SYT1, in the regenerating retinal RGC-5 cell line [87]. The perinatal dietary deficiency of ω-3 PUFA was reported to lead to the down-regulation of the SYT1 and SYP proteins in hippocampal synapses [88], and remarkably, ω-3 PUFA supplementation increased SYP protein expression in the hippocampus, resulting in increased synaptogenesis and neurogenesis [89,90]. In our current study, the potential neuroprotective effects conferred by ω-3 PUFA on the neuro-vascular retinal tissue were further substantiated by the significant up-regulation of both proteins exclusively in both supplemented groups. Synaptophysin, which was highly expressed in the ω-3 PUFA-administered floxed mice, is an integral presynaptic membrane protein essential for visual signal transmission [91], and hence, is widely used as a marker for synaptic plasticity in the brain [89]. On one hand, the differential expression of Syp, amongst others, together with the significant regulation of canonical pathways related to neurotransmission, synaptogenesis and synaptic functions in the floxed group highly reflect the preservation of neuronal functions in the retina attributed to ω-3 PUFA in normal mice. On the other hand, the collective findings in the KO mice formulated an intriguing connection between the retinal degenerative activities in Cyp2c44-deficient mice and heightened regenerative processes at the proteome level to restore retinal functions following ω-3 PUFA intake in this genotype.

The retina is a unique ocular tissue, which encompasses different neuronal and vascular networks. Therefore, any deleterious changes are often reflected in the regulation of elaborate mechanisms that strive to preserve critical homeostasis between vascular supply and the demands of neuronal energy requirements. It is a prevailing dogma that vascular remodelling is intimately related to retinal bioenergetics, and hence, is an early reflection of subjacent changes in the retinal neuronal metabolism [30,92]. In the present investigation, the proteome vicissitudes hinted at retinal remodelling in the KO group, as demonstrated by the expression of proteins implicated in retinal architecture maintenance, such as Rs1 and Kif21a, as well as by the inhibition of microtubule dynamics and cytoskeleton organizational functions. The former protein was up-regulated in the KO retina, a phenomenon that was also observed in a murine model of retinal detachment consistent with its role in maintaining photoreceptor stability and participation in cytoskeleton organization [93,94]. Meanwhile, the microtubule-dependent molecular motor protein Kif21a was up-regulated in the KO group but was significantly down-regulated following ω-3 PUFA intake. This expression profile fits aptly to the correlation of increased Kif21a expression to axonal guidance abnormalities and neurodegenerative diseases [95,96]. An up-regulation of this protein was also shown to have an inhibitory effect on the microtubule-associated protein 1b (Map 1b) [97], which was also identified in our study, albeit not differentially expressed. The ‘microtubule hypothesis of glaucoma’ denotes that the disruption of microtubules precedes the loss of retinal ganglion cell axons [98], which can be delineated in part to the proteome changes in the KO retina observed in our study.

The ω-3 PUFAs are known to modify the physicochemical properties of the cell membrane, thereby altering the membrane fluidity to facilitate various downstream signal transduction functions *via* a myriad of transcription factors [53,99,100]. Two such upstream regulators, which were also found to be involved in the regulation of ophthalmic arterial proteins comprising TGF-β1 and PPARs, were significantly implicated in the regulation of retinal proteins. However, unlike the artery in which the former was activated at different magnitudes in both KO groups, it was inhibited in the KO group and activated in the ω-3 PUFA-fed KO group in the retina. This paradoxical result can be traced back to the inherent attribute of TGF-β1 that can act as both an inhibitory and promoting factor [69]. The loss of TGF-β1 signalling components leads to compromised microvessel integrity and defective vascular structure [69,74]. A study by Walshe et al. elegantly confirmed the existence of a constitutive TGF-β signalling in murine retina that is essential for retinal vascular and neural cell survival [74]. Two different PPAR isoforms were predicted to be significantly involved in the regulation of proteins in both ω-3 PUFA-fed groups, in addition to the predicted inhibition of NF-κB, a well-known transcription factor related to inflammatory responses [22,101] in the supplemented floxed mice. These findings are not surprising, as the protective effects of ω-3 PUFA and their metabolites are known to be largely mediated *via* the activation of PPAR [102] and suppression of NF-κB [61,101,103,104].

It is important to note the main limitation of this study. We acknowledge that the major findings were not validated by baroque orthogonal methods. However, the main aim of this study was to first establish the feasibility of an acute ω-3 PUFA supplementation by determining whether there were significant effects manifested at the proteome level within a very short time compared to the existing studies, and second, to explore the potential changes attributed to the *Cyp2c44* gene knockout, because it is known that other CYP isoforms readily compensate for the loss of activity of one subfamily [105]. It is also noteworthy that the retinal samples were analysed individually to substantiate the results in an unbiased manner, which further strengthens the validity of our findings. Importantly, floxed littermates were used as controls in our study to ensure the rigor and reproducibility of our data using multiparous animals [106,107]. Nevertheless, the promising pilot outcomes obtained from this investigation have opened exciting avenues for future work to elucidate the mechanistic underpinnings of ω-3 PUFA, in order to harness its inherent therapeutic potential in the eye.

## 5. Conclusions

In conclusion, our study provides the first in-depth data supporting acute beneficial proteome changes underlying dietary supplementation with ω-3 PUFA in two different ocular vascular beds within a very short period of 7 days. Importantly, we elucidated the mechanisms that govern the vascular and retinal functionalities in the absence of the main CYP isoform using a specific *Cyp2c44* gene-deleted mouse model. These results collectively highlighted that the global *Cyp2c44* gene-knockout resulted in more profound deleterious alterations in the retina compared to the ophthalmic artery, which is reminiscent of our retrospective study comparing both ocular tissues [30] and supported our current hypothesis. Remarkably, ω-3 PUFA intake was shown to abrogate early signs of degenerative mechanisms in the retina by restoring several key metabolic functions in the KO mice, while enhancing the neuronal and synaptic transmission in the control floxed mice. Conversely, although the effects of the gene KO were not as apparent in the ophthalmic artery, there were distinct proteome changes that supported its role in the maintenance and preservation of vascular integrity in both genotypes. Taken together, the findings emerging from our investigation advance the mechanistic understanding of the vaso- and neuronal-protective effects conferred by ω-3 PUFA, which can be a promising basis for adjunct therapy for ophthalmic pathologies owing to its fast action at the proteome level.

## Data Availability

The data presented in this study are available in this published article and its Supplementary Materials Files.

## Supplementary Materials

The following supporting information can be downloaded at: https://www.mdpi.com/article/10.3390/cells11213494/s1, Figure S1: Body weight profile of Cyp2c44 KO and littermate floxed mice (Cyp2c44 ^fl/fl^) before (0) and after (day 8) daily oral gavage for 7 days with either omega 3 PUFA (ω-3) or PBS; Figure S2: Venn diagrams depicting the significantly differentially expressed proteins (*p* < 0.05) identified in the (a) ophthalmic artery and (b) retina of Cyp2c44 KO (KO) and littermate floxed mice (fl) following daily oral gavage for 7 days with either omega 3 PUFA (ω3) or PBS; Figure S3: Protein–protein interaction (PPI) network of the differentially expressed ophthalmic arterial proteins of the floxed mice following daily oral gavage for 7 days with either omega 3 PUFA (ω3) or PBS (fl ω3/fl PBS group); Figure S4: Gene heatmaps of selected diseases and biological functions in the ophthalmic artery; Figure S5: Protein–protein interaction (PPI) network of the differentially expressed retinal proteins of the floxed mice following daily oral gavage for 7 days with either omega 3 PUFA (ω3) or PBS (fl ω3/fl PBS); Figure S6: Gene heatmaps of selected diseases and biological functions in the retina; Table S1: MaxQuant parameters for ophthalmic artery and retina; Table S2: Total proteins identified from the ophthalmic artery; Table S3: Total retinal proteins; Table S4: Differentially expressed proteins identified from the ophthalmic artery; Table S5: Differentially expressed retinal proteins.

## References

[1] Gong Y., Fu Z., Liegl R., Chen J., Hellström A., Smith L.E. (2017). ω-3 and ω-6 long-chain PUFAs and their enzymatic metabolites in neovascular eye diseases. Am. J. Clin. Nutr..

[2] Prokopiou E., Kolovos P., Georgiou C., Kalogerou M., Potamiti L., Sokratous K., Kyriacou K., Georgiou T. (2019). Omega-3 fatty acids supplementation protects the retina from age-associated degeneration in aged C57BL/6J mice. BMJ Open Ophthalmol..

[3] Prokopiou E., Kolovos P., Kalogerou M., Neokleous A., Papagregoriou G., Deltas C., Malas S., Georgiou T. (2017). Therapeutic potential of omega-3 fatty acids supplementation in a mouse model of dry macular degeneration. BMJ Open Ophthalmol..

[4] Tourtas T., Birke M.T., Kruse F.E., Welge-Lüssen U.-C., Birke K. (2012). Preventive effects of omega-3 and omega-6 Fatty acids on peroxide mediated oxidative stress responses in primary human trabecular meshwork cells. PLoS ONE.

[5] Arterburn L.M., Hall E.B., Oken H. (2006). Distribution, interconversion, and dose response of n − 3 fatty acids in humans. Am. J. Clin. Nutr..

[6] Bush R.A., Malnoe A., Reme C.E., Williams T.P. (1994). Dietary deficiency of N-3 fatty acids alters rhodopsin content and function in the rat retina. Investig. Ophthalmol. Vis. Sci..

[7] Stinson A.M., Wiegand R., Anderson R. (1991). Recycling of docosahexaenoic acid in rat retinas during n-3 fatty acid deficiency. J. Lipid Res..

[8] Downie L.E., Vingrys A.J. (2018). Oral omega-3 supplementation lowers intraocular pressure in normotensive adults. Transl. Vis. Sci. Technol..

[9] Nguyen C.T., Bui B.V., Sinclair A.J., Vingrys A.J. (2007). Dietary omega 3 fatty acids decrease intraocular pressure with age by increasing aqueous outflow. Investig. Ophthalmol. Vis. Sci..

[10] Schnebelen C., Pasquis B., Salinas-Navarro M., Joffre C., Creuzot-Garcher C.P., Vidal-Sanz M., Bron A.M., Bretillon L., Acar N. (2009). A dietary combination of omega-3 and omega-6 polyunsaturated fatty acids is more efficient than single supplementations in the prevention of retinal damage induced by elevation of intraocular pressure in rats. Graefe’s Arch. Clin. Exp. Ophthalmol..

[11] Huang W.-B., Fan Q., Zhang X.-L. (2011). Cod liver oil: A potential protective supplement for human glaucoma. Int. J. Ophthalmol..

[12] Christen W.G., Schaumberg D.A., Glynn R.J., Buring J.E. (2011). Dietary ω-3 fatty acid and fish intake and incident age-related macular degeneration in women. Arch. Ophthalmol..

[13] Dawczynski J., Jentsch S., Schweitzer D., Hammer M., Lang G.E., Strobel J. (2013). Long term effects of lutein, zeaxanthin and omega-3-LCPUFAs supplementation on optical density of macular pigment in AMD patients: The LUTEGA study. Graefe’s Arch. Clin. Exp. Ophthalmol..

[14] García-Layana A., Recalde S., Alamán A.S., Robredo P.F. (2013). Effects of lutein and docosahexaenoic acid supplementation on macular pigment optical density in a randomized controlled trial. Nutrients.

[15] Murayama K., Yoneya S., Miyauchi O., Adachi-Usami E., Nishikawa M. (2002). Fish oil (polyunsaturated fatty acid) prevents ischemic-induced injury in the mammalian retina. Exp. Eye Res..

[16] Georgiou T., Wen Y.-T., Chang C.-H., Kolovos P., Kalogerou M., Prokopiou E., Neokleous A., Huang C.-T., Tsai R.-K. (2017). Neuroprotective effects of omega-3 polyunsaturated fatty acids in a rat model of anterior ischemic optic neuropathy. Investig. Ophthalmol. Vis. Sci..

[17] Tan J.S., Wang J.J., Flood V., Mitchell P. (2009). Dietary fatty acids and the 10-year incidence of age-related macular degeneration: The Blue Mountains Eye Study. Arch. Ophthalmol..

[18] Ho L., van Leeuwen R., Witteman J.C., van Duijn C.M., Uitterlinden A.G., Hofman A., de Jong P.T., Vingerling J.R., Klaver C.C. (2011). Reducing the genetic risk of age-related macular degeneration with dietary antioxidants, zinc, and ω-3 fatty acids: The Rotterdam study. Arch. Ophthalmol..

[19] Querques G., Benlian P., Chanu B., Portal C., Coscas G., Soubrane G., Souied E. (2009). Nutritional AMD treatment phase I (NAT-1): Feasibility of oral DHA supplementation in age-related macular degeneration. Eur. J. Ophthalmol..

[20] Sala-Vila A., Díaz-López A., Valls-Pedret C., Cofán M., García-Layana A., Lamuela-Raventós R.-M., Castañer O., Zanon-Moreno V., Martinez-Gonzalez M.A., Toledo E. (2016). Dietary marine ω-3 fatty acids and incident sight-threatening retinopathy in middle-aged and older individuals with type 2 diabetes: Prospective investigation from the PREDIMED trial. JAMA Ophthalmol..

[21] Arnold C., Markovic M., Blossey K., Wallukat G., Fischer R., Dechend R., onkel A., von Schacky C., Luft F.C., Muller D.N. (2010). Arachidonic acid-metabolizing cytochrome P450 enzymes are targets of ω-3 fatty acids. J. Biol. Chem..

[22] Méndez L., Ciordia S., Fernández M.S., Juárez S., Ramos A., Pazos M., Gallardo J.M., Torres J.L., Nogués M.R., Medina I. (2017). Changes in liver proteins of rats fed standard and high-fat and sucrose diets induced by fish omega-3 PUFAs and their combination with grape polyphenols according to quantitative proteomics. J. Nutr. Biochem..

[23] Yanai R., Mulki L., Hasegawa E., Takeuchi K., Sweigard H., Suzuki J., Gaissert P., Vavvas D.G., Sonoda K.-H., Rothe M. (2014). Cytochrome P450-generated metabolites derived from ω-3 fatty acids attenuate neovascularization. Proc. Natl. Acad. Sci. USA.

[24] Amirmokhtari N., Foresi B.D., Dewan S.S., Bouhenni R.A., Smith M.A. (2021). Absence of cytochrome P450-1b1 increases susceptibility of pressure-induced axonopathy in the murine retinal projection. Front. Cell Dev. Biol..

[25] Capozzi M.E., McCollum G.W., Penn J.S. (2014). The role of cytochrome P450 epoxygenases in retinal angiogenesis. Investig. Ophthalmol. Vis. Sci..

[26] Hu J., Geyer A., Dziumbla S., Awwad K., Zeldin D.C., Schunck W.-H., Popp R., Frömel T., Fleming I. (2017). Role of Müller cell cytochrome P450 2c44 in murine retinal angiogenesis. Prostaglandins Other Lipid Mediat..

[27] Shao Z., Fu Z., Stahl A., Joyal J.-S., Hatton C., Juan A., Hurst C., Evans L., Cui Z., Pei D. (2014). Cytochrome P450 2C8 ω3-long-chain polyunsaturated fatty acid metabolites increase mouse retinal pathologic neovascularization—Brief report. Arterioscler.Thromb. Vasc. Biol..

[28] Hu J., Sisignano M., Brecht R., Perumal N., Angioni C., Bibli I.-S., Fisslthaler B., Kleinert H., Pfeiffer N., Fleming I. (2021). Cyp2c44 epoxygenase-derived epoxyeicosatrienoic acids in vascular smooth muscle cells elicit vasoconstriction of the murine ophthalmic artery. Sci. Rep..

[29] Manicam C., Staubitz J., Brochhausen C., Grus F.H., Pfeiffer N., Gericke A. (2016). The gatekeepers in the mouse ophthalmic artery: Endothelium-dependent mechanisms of cholinergic vasodilation. Sci. Rep..

[30] Perumal N., Straßburger L., Herzog D.P., Müller M.B., Pfeiffer N., Grus F.H., Manicam C. (2020). Bioenergetic shift and actin cytoskeleton remodelling as acute vascular adaptive mechanisms to angiotensin II in murine retina and ophthalmic artery. Redox Biol..

[31] Perumal N., Straßburger L., Schmelter C., Gericke A., Pfeiffer N., Grus F.H., Manicam C. (2019). Sample preparation for mass-spectrometry-based proteomics analysis of ocular microvessels. JoVE (J. Vis. Exp.).

[32] Cox J., Mann M. (2008). MaxQuant enables high peptide identification rates, individualized ppb-range mass accuracies and proteome-wide protein quantification. Nat. Biotechnol..

[33] Cox J., Neuhauser N., Michalski A., Scheltema R.A., Olsen J.V., Mann M. (2011). Andromeda: A peptide search engine integrated into the MaxQuant environment. J. Proteome Res..

[34] Tyanova S., Temu T., Cox J. (2016). The MaxQuant computational platform for mass spectrometry-based shotgun proteomics. Nat. Protoc..

[35] Krämer A., Green J., Pollard J., Tugendreich S. (2014). Causal analysis approaches in ingenuity pathway analysis. Bioinformatics.

[36] Alhaider A.A., Bayoumy N., Argo E., Gader A.G., Stead D.A. (2012). Survey of the camel urinary proteome by shotgun proteomics using a multiple database search strategy. Proteomics.

[37] Alpi E., Griss J., da Silva A.W.S., Bely B., Antunes R., Zellner H., Ríos D., O’Donovan C., Vizcaíno J.A., Martin M.J. (2015). Analysis of the tryptic search space in UniProt databases. Proteomics.

[38] Shanmugam A.K., Nesvizhskii A.I. (2015). Effective leveraging of targeted search spaces for improving peptide identification in tandem mass spectrometry based proteomics. J. Proteome Res..

[39] Tanca A., Palomba A., Fraumene C., Pagnozzi D., Manghina V., Deligios M., Muth T., Rapp E., Martens L., Addis M.F. (2016). The impact of sequence database choice on metaproteomic results in gut microbiota studies. Microbiome.

[40] Prokopiou E., Kolovos P., Kalogerou M., Neokleous A., Nicolaou O., Sokratous K., Kyriacou K., Georgiou T. (2018). Omega-3 fatty acids supplementation: Therapeutic potential in a mouse model of Stargardt disease. Investig. Ophthalmol. Vis. Sci..

[41] Pan M., Zhao F., Xie B., Wu H., Zhang S., Ye C., Guan Z., Kang L., Zhang Y., Zhou X. (2021). Dietary ω-3 polyunsaturated fatty acids are protective for myopia. Proc. Natl. Acad. Sci. USA.

[42] Ahmad Sopian N.F., Ajat M., Shafie N.I., Mohd Noor M.H., Ebrahimi M., Rajion M.A., Meng G.Y., Ahmad H. (2015). Does short-term dietary omega-3 fatty acid supplementation influence brain hippocampus gene expression of zinc transporter-3?. Int. J. Mol. Sci..

[43] Ginty A.T., Conklin S.M. (2015). Short-term supplementation of acute long-chain omega-3 polyunsaturated fatty acids may alter depression status and decrease symptomology among young adults with depression: A preliminary randomized and placebo controlled trial. Psychiatry Res..

[44] Grenon S.M., Owens C.D., Nosova E.V., Hughes-Fulford M., Alley H.F., Chong K., Perez S., Yen P.K., Boscardin J., Hellmann J. (2015). Short-Term, High-Dose Fish Oil Supplementation Increases the Production of Omega-3 Fatty Acid–Derived Mediators in Patients with Peripheral Artery Disease (the OMEGA-PAD I Trial). J. Am. Heart Assoc..

[45] Bhargava R., Kumar P., Arora Y. (2016). Short-term omega 3 fatty acids treatment for dry eye in young and middle-aged visual display terminal users. Eye Contact Lens Sci. Clin. Pract..

[46] Li F., Chen H., Anderson R.E. (2001). Biosynthesis of docosahexaenoate-containing glycerolipid molecular species in the retina. J. Mol. Neurosci..

[47] Allam-Ndoul B., Guénard F., Barbier O., Vohl M.-C. (2017). Effect of different concentrations of omega-3 fatty acids on stimulated THP-1 macrophages. Genes Nutr..

[48] Bradberry J.C., Hilleman D.E. (2013). Overview of omega-3 fatty acid therapies. Pharm. Ther..

[49] Behrendt L., Kurth I., Kaether C. (2019). A disease causing ATLASTIN 3 mutation affects multiple endoplasmic reticulum-related pathways. Cell. Mol. Life Sci..

[50] Lee J.E., Yuan H., Liang F.-X., Sehgal P.B. (2013). Nitric oxide scavenging causes remodeling of the endoplasmic reticulum, Golgi apparatus and mitochondria in pulmonary arterial endothelial cells. Nitric Oxide.

[51] Lü L., Niu L., Hu J. (2020). “At last in” the physiological roles of the tubular ER network. Biophys. Rep..

[52] Cao Z., Hao Y., Fung C.W., Lee Y.Y., Wang P., Li X., Xie K., Lam W.J., Qiu Y., Tang B.Z. (2019). Dietary fatty acids promote lipid droplet diversity through seipin enrichment in an ER subdomain. Nat. Commun..

[53] López D., Orta X., Casós K., Sáiz M.P., Puig-Parellada P., Farriol M., Mitjavila M.T. (2004). Upregulation of endothelial nitric oxide synthase in rat aorta after ingestion of fish oil-rich diet. Am. J. Physiol. -Heart Circ. Physiol..

[54] Leung S., Sum W., Shi Y. (2022). The glycolytic process in endothelial cells and its implications. Acta Pharmacol. Sin..

[55] Zhang R., Shen W., Du J., Gillies M.C. (2020). Selective knockdown of hexokinase 2 in rods leads to age-related photoreceptor degeneration and retinal metabolic remodeling. Cell Death Dis..

[56] Sullivan L.S., Koboldt D.C., Bowne S.J., Lang S., Blanton S.H., Cadena E., Avery C.E., Lewis R.A., Webb-Jones K., Wheaton D.H. (2014). A dominant mutation in hexokinase 1 (HK1) causes retinitis pigmentosa. Investig. Ophthalmol. Vis. Sci..

[57] Nederlof R., Eerbeek O., Hollmann M.W., Southworth R., Zuurbier C.J. (2014). Targeting hexokinase II to mitochondria to modulate energy metabolism and reduce ischaemia-reperfusion injury in heart. Br. J. Pharmacol..

[58] Zhang W.-h., Qiu M.-h., Wang X.-j., Sun K., Zheng Y., Jing Z.-c. (2014). Up-regulation of hexokinase1 in the right ventricle of monocrotaline induced pulmonary hypertension. Respir. Res..

[59] Zhao M., Wang Q., Liu L., Geng T., Gong D. (2022). Mitochondrial-bound hexokinase 1 can affect the glucolipid metabolism and reactive oxygen species production in goose fatty liver. Ital. J. Anim. Sci..

[60] Flachsbart F., Ufer M., Kleindorp R., Nikolaus S., Schreiber S., Nebel A. (2011). Genetic variation in the CYP2C monooxygenase enzyme subfamily shows no association with longevity in a German population. J. Gerontol. Ser. A Biomed. Sci. Med. Sci..

[61] Wendel M., Heller A.R. (2009). Anticancer actions of omega-3 fatty acids-current state and future perspectives. Anti-Cancer Agents Med. Chem. (Former. Curr. Med. Chem.-Anti-Cancer Agents).

[62] Pearsall E.A., Cheng R., Zhou K., Takahashi Y., Matlock H.G., Vadvalkar S.S., Shin Y., Fredrick T.W., Gantner M.L., Meng S. (2017). PPARα is essential for retinal lipid metabolism and neuronal survival. BMC Biol..

[63] Steneberg P., Rubins N., Bartoov-Shifman R., Walker M.D., Edlund H. (2005). The FFA receptor GPR40 links hyperinsulinemia, hepatic steatosis, and impaired glucose homeostasis in mouse. Cell Metab..

[64] Lefebvre P., Chinetti G., Fruchart J.-C., Staels B. (2006). Sorting out the roles of PPARα in energy metabolism and vascular homeostasis. J. Clin. Investig..

[65] Finck B.N., Lehman J.J., Leone T.C., Welch M.J., Bennett M.J., Kovacs A., Han X., Gross R.W., Kozak R., Lopaschuk G.D. (2002). The cardiac phenotype induced by PPARα overexpression mimics that caused by diabetes mellitus. J. Clin. Investig..

[66] Kalucka J., Bierhansl L., Conchinha N.V., Missiaen R., Elia I., Brüning U., Scheinok S., Treps L., Cantelmo A.R., Dubois C. (2018). Quiescent endothelial cells upregulate fatty acid β-oxidation for vasculoprotection via redox homeostasis. Cell Metab..

[67] Patella F., Schug Z.T., Persi E., Neilson L.J., Erami Z., Avanzato D., Maione F., Hernandez-Fernaud J.R., Mackay G., Zheng L. (2015). Proteomics-Based Metabolic Modeling Reveals That Fatty Acid Oxidation (FAO) Controls Endothelial Cell (EC) Permeability. Mol. Cell. Proteom..

[68] Manicam C., Ginter N., Li H., Xia N., Goloborodko E., Zadeh J.K., Musayeva A., Pfeiffer N., Gericke A. (2017). Compensatory vasodilator mechanisms in the ophthalmic artery of endothelial nitric oxide synthase gene knockout mice. Sci. Rep..

[69] Goumans M.-J., Liu Z., Ten Dijke P. (2009). TGF-β signaling in vascular biology and dysfunction. Cell Res..

[70] Ding R., Darland D.C., Parmacek M.S., D’amore P.A. (2004). Endothelial–mesenchymal interactions in vitro reveal molecular mechanisms of smooth muscle/pericyte differentiation. Stem Cells Dev..

[71] Gordon K.J., Blobe G.C. (2008). Role of transforming growth factor-β superfamily signaling pathways in human disease. Biochim. Et Biophys. Acta (BBA)-Mol. Basis Dis..

[72] Li H., Wallerath T., Förstermann U. (2002). Physiological mechanisms regulating the expression of endothelial-type NO synthase. Nitric Oxide.

[73] Ten Dijke P., Arthur H.M. (2007). Extracellular control of TGFβ signalling in vascular development and disease. Nat. Rev. Mol. Cell Biol..

[74] Walshe T.E., Saint-Geniez M., Maharaj A.S., Sekiyama E., Maldonado A.E., D’Amore P.A. (2009). TGF-β is required for vascular barrier function, endothelial survival and homeostasis of the adult microvasculature. PLoS ONE.

[75] Rey S., Semenza G.L. (2010). Hypoxia-inducible factor-1-dependent mechanisms of vascularization and vascular remodelling. Cardiovasc. Res..

[76] Manalo D.J., Rowan A., Lavoie T., Natarajan L., Kelly B.D., Ye S.Q., Garcia J.G., Semenza G.L. (2005). Transcriptional regulation of vascular endothelial cell responses to hypoxia by HIF-1. Blood.

[77] Geraci M.W., Gao B., Shepherd D.C., Moore M.D., Westcott J.Y., Fagan K.A., Alger L.A., Tuder R.M., Voelkel N.F. (1999). Pulmonary prostacyclin synthase overexpression in transgenic mice protects against development of hypoxic pulmonary hypertension. J. Clin. Investig..

[78] McCullough K.T., Boye S.L., Fajardo D., Calabro K., Peterson J.J., Strang C.E., Chakraborty D., Gloskowski S., Haskett S., Samuelsson S. (2019). Somatic gene editing of GUCY2D by AAV-CRISPR/Cas9 alters retinal structure and function in mouse and macaque. Hum. Gene Ther..

[79] Mihelec M., Pearson R.A., Robbie S.J., Buch P.K., Azam S.A., Bainbridge J.W., Smith A.J., Ali R.R. (2011). Long-term preservation of cones and improvement in visual function following gene therapy in a mouse model of leber congenital amaurosis caused by guanylate cyclase-1 deficiency. Hum. Gene Ther..

[80] Lowe D.G., Dizhoor A.M., Liu K., Gu Q., Spencer M., Laura R., Lu L., Hurley J.B. (1995). Cloning and expression of a second photoreceptor-specific membrane retina guanylyl cyclase (RetGC), RetGC-2. Proc. Natl. Acad. Sci. USA.

[81] Baehr W., Karan S., Maeda T., Luo D.-G., Li S., Bronson J.D., Watt C.B., Yau K.-W., Frederick J.M., Palczewski K. (2007). The function of guanylate cyclase 1 and guanylate cyclase 2 in rod and cone photoreceptors. J. Biol. Chem..

[82] Lakowski J., Baron M., Bainbridge J., Barber A., Pearson R., Ali R., Sowden J. (2010). Cone and rod photoreceptor transplantation in models of the childhood retinopathy Leber congenital amaurosis using flow-sorted Crx-positive donor cells. Hum. Mol. Genet..

[83] Beetz N., Hein L. (2011). The physiological roles of phosducin: From retinal function to stress-dependent hypertension. Cell. Mol. Life Sci..

[84] Cheng C.L., Molday R.S. (2013). Changes in gene expression associated with retinal degeneration in the rd3 mouse. Mol. Vis..

[85] Georgiou T., Prokopiou E. (2015). The new era of omega-3 fatty acids supplementation: Therapeutic effects on dry age-related macular degeneration. J. Stem Cells.

[86] Böhm M.R., Pfrommer S., Chiwitt C., Brückner M., Melkonyan H., Thanos S. (2012). Crystallin-β-b2-overexpressing NPCs support the survival of injured retinal ganglion cells and photoreceptors in rats. Investig. Ophthalmol. Vis. Sci..

[87] Liedtke T., Schwamborn J.C., Schröer U., Thanos S. (2007). Elongation of axons during regeneration involves retinal crystallin β b2 (crybb2). Mol. Cell. Proteom..

[88] Aryal S., Hussain S., Drevon C.A., Nagelhus E., Hvalby Ø., Jensen V., Walaas S.I., Davanger S. (2019). Omega-3 fatty acids regulate plasticity in distinct hippocampal glutamatergic synapses. Eur. J. Neurosci..

[89] Hajjar T., Goh Y.M., Rajion M.A., Vidyadaran S., Li T.A., Ebrahimi M. (2013). Alterations in neuronal morphology and synaptophysin expression in the rat brain as a result of changes in dietary n-6: N-3 fatty acid ratios. Lipids Health Dis..

[90] Su H.-M. (2010). Mechanisms of n-3 fatty acid-mediated development and maintenance of learning memory performance. J. Nutr. Biochem..

[91] Dan C., Jian-Bin T., Hui W., Le-Ping Z., Jin Z., Ju-Fang H., Xue-Gang L. (2008). Synaptophysin expression in rat retina following acute high intraocular pressure. Acta Histochem. Cytochem..

[92] Joyal J.-S., Gantner M.L., Smith L.E. (2018). Retinal energy demands control vascular supply of the retina in development and disease: The role of neuronal lipid and glucose metabolism. Prog. Retin. Eye Res..

[93] Gehrig A., Langmann T., Horling F., Janssen A., Bonin M., Walter M., Poths S., Weber B.H. (2007). Genome-wide expression profiling of the retinoschisin-deficient retina in early postnatal mouse development. Investig. Ophthalmol. Vis. Sci..

[94] Kjellström S., Ghosh F., Vijayasarathy C., Andréasson S. (2014). Alteration of Vitreal Retinoschisin Level in Human Primary Retinal Detachment. JAMA Ophthalmol..

[95] Bianchi S., van Riel W.E., Kraatz S.H., Olieric N., Frey D., Katrukha E.A., Jaussi R., Missimer J., Grigoriev I., Olieric V. (2016). Structural basis for misregulation of kinesin KIF21A autoinhibition by CFEOM1 disease mutations. Sci. Rep..

[96] Morfini G.A., Burns M., Binder L.I., Kanaan N.M., LaPointe N., Bosco D.A., Brown R.H., Brown H., Tiwari A., Hayward L. (2009). Axonal transport defects in neurodegenerative diseases. J. Neurosci..

[97] Vivian A.J. (2020). Congenital fibrosis of the extra-ocular muscles (CFEOM) and the cranial dysinnervation disorders. Eye.

[98] Sharoukhov D., Bucinca-Cupallari F., Lim H. (2018). Microtubule imaging reveals cytoskeletal deficit predisposing the retinal ganglion cell axons to atrophy in DBA/2J. Investig. Ophthalmol. Vis. Sci..

[99] Sherratt S.C., Dawoud H., Bhatt D.L., Malinski T., Mason R.P. (2021). Omega-3 and omega-6 fatty acids have distinct effects on endothelial fatty acid content and nitric oxide bioavailability. Prostaglandins Leukot. Essent. Fat. Acids.

[100] Martins M.A., Moss M.B., Mendes I.K., Águila M.B., Mandarim-de-Lacerda C.A., Brunini T.M., Mendes-Ribeiro A.C. (2014). Role of dietary fish oil on nitric oxide synthase activity and oxidative status in mice red blood cells. Food Funct..

[101] Felau S.M., Sales L.P., Solis M.Y., Hayashi A.P., Roschel H., Sá-Pinto A.L. (2018). Omega-3 fatty acid supplementation improves endothelial function in primary antiphospholipid syndrome: A small-scale randomized double-blind placebo-controlled trial. Front. Immunol..

[102] Stahl A., Sapieha P., Connor K.M., SanGiovanni J.P., Chen J., Aderman C.M. (2010). PPARγ mediates a direct antiangiogenic effect of ω3-PUFAs in proliferative retinopathy. Circ. Res..

[103] Delbosc S., Glorian M., Le Port A.-S., Béréziat G., Andréani M., Limon I. (2008). The benefit of docosahexanoic acid on the migration of vascular smooth muscle cells is partially dependent on Notch regulation of MMP-2/-9. Am. J. Pathol..

[104] Hu S., Bae M., Park Y.-K., Lee J.-Y. (2020). n-3 PUFAs inhibit TGFβ1-induced profibrogenic gene expression by ameliorating the repression of PPARγ in hepatic stellate cells. J. Nutr. Biochem..

[105] Holla V.R., Adas F., Imig J.D., Zhao X., Price E., Olsen N. (2001). Alterations in the regulation of androgen-sensitive Cyp 4a monooxygenases cause hypertension. Proc. Natl. Acad. Sci. USA.

[106] Holmdahl R., Malissen B. (2012). The Need for Littermate Controls.

[107] Jiménez J.A., Zylka M.J. (2021). Controlling litter effects to enhance rigor and reproducibility with rodent models of neurodevelopmental disorders. J. Neurodev. Disord..

