# Supplementary material for: Short-Term Omega-3 Supplementation Modulates Novel Neurovascular and Fatty Acid Metabolic Proteome Changes in the Retina and Ophthalmic Artery of Mice with Targeted Cyp2c44 Gene Deletion
Source: Cells. 2022 Nov 4;11(21):3494. doi: 10.3390/cells11213494 (PMC9658563; doi:10.3390/cells11213494)
Supplement: Supplementary file 1 [file cells-11-03494-s001.zip › Supplementary Figures_Perumal et al 2022_Cells.pdf]

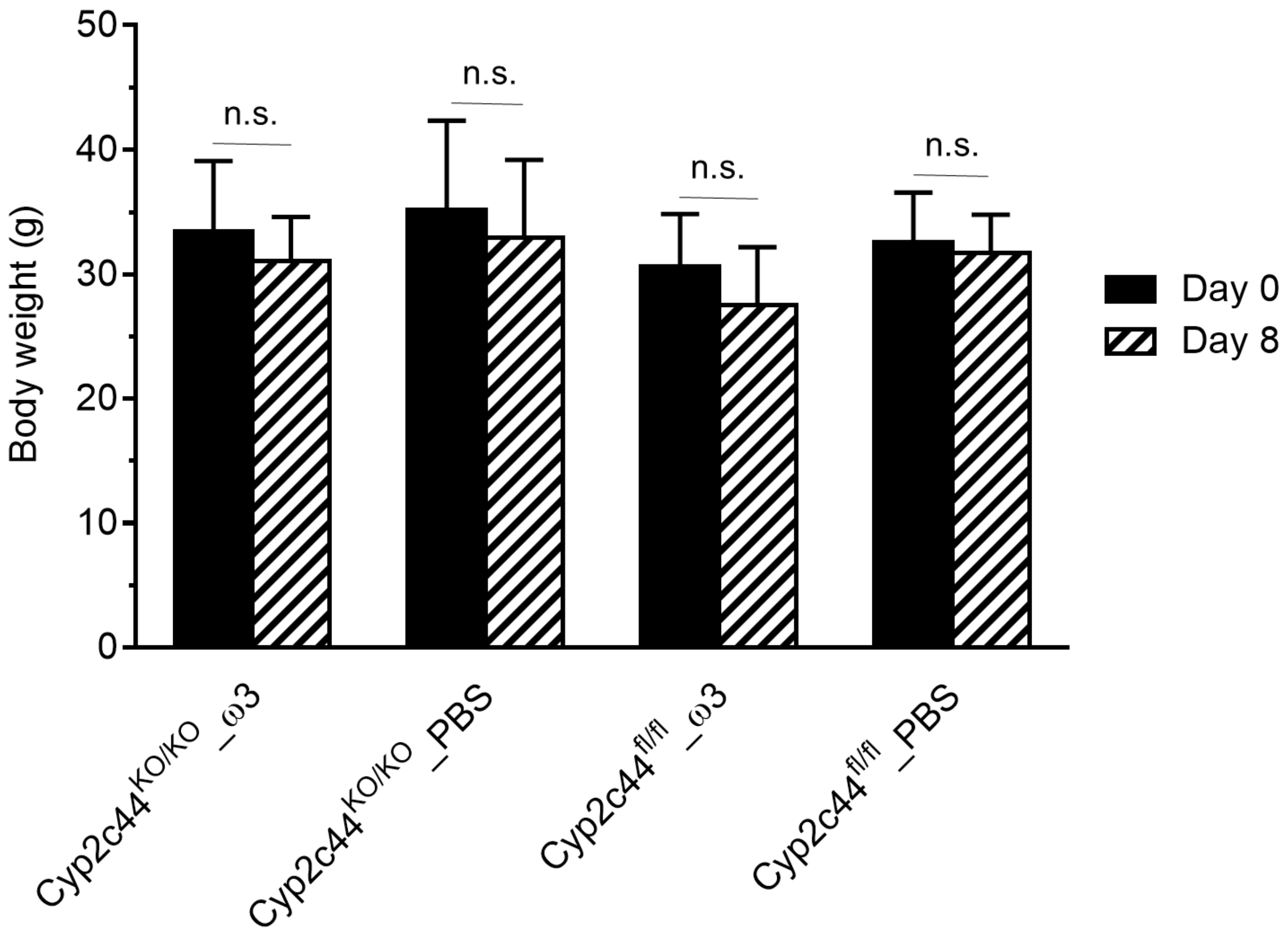

Fig. S1: Body weight profile of Cyp2c44 KO and littermate floxed mice (Cyp2c44 $^{fl/fl}$) before (0) and after (day 8) daily oral gavage for 7 days with either omega 3 PUFA (ω-3) or PBS. Bars represent the mean ± s.e.m., n = 9/ group. n.s.: non-significant ($p > 0.05$).

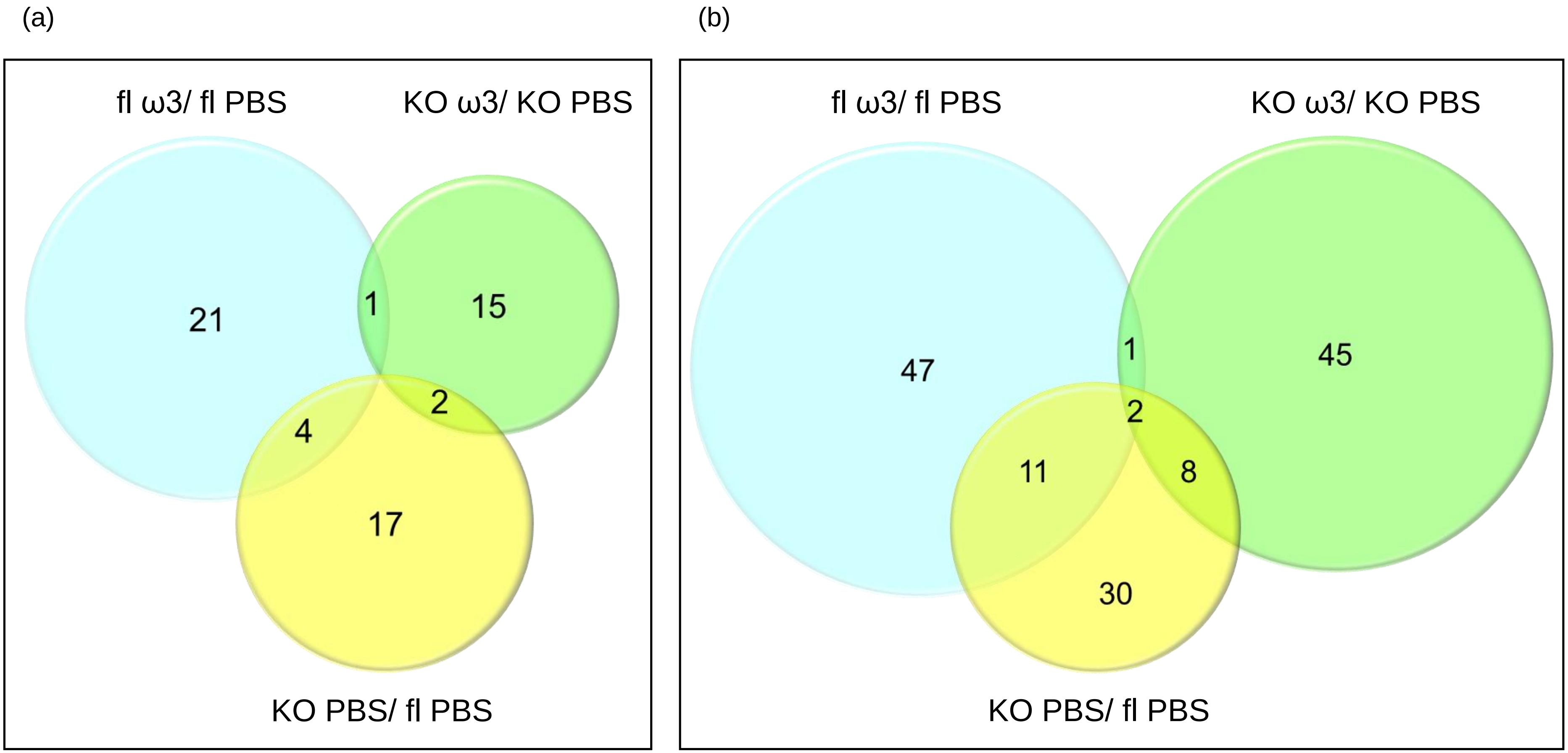

Fig. S2: Venn diagrams depicting the significantly differentially expressed proteins (p < 0.05) identified in the (a) ophthalmic artery and (b) retina of Cyp2c44 KO (KO) and littermate floxed mice (fl) following daily oral gavage for 7 days with either omega 3 PUFA (ω3) or PBS.

(a)

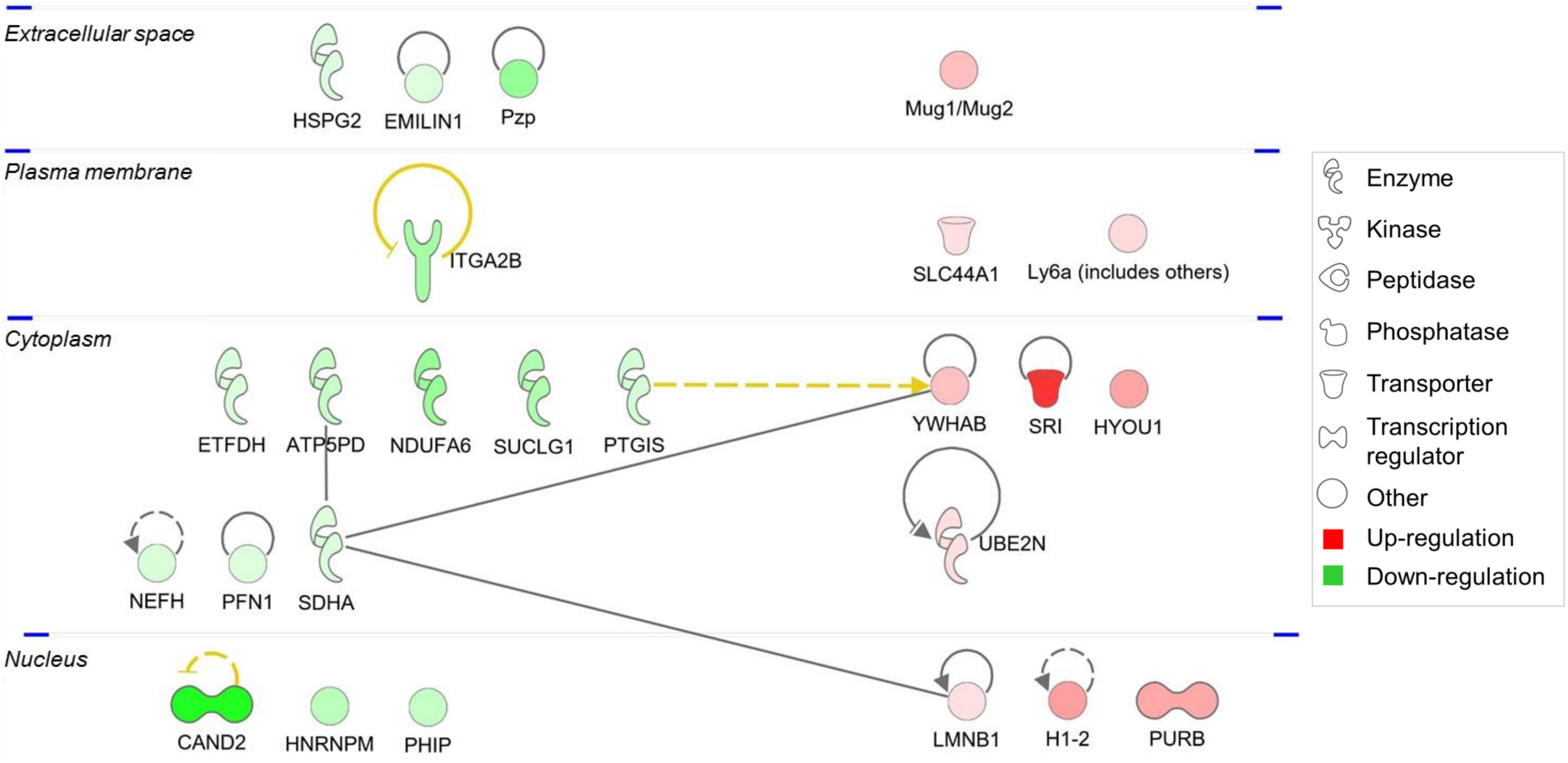

Fig. S3: Protein-protein interaction (PPI) network of the differentially expressed ophthalmic arterial proteins of the floxed mice following daily oral gavage for 7 days with either omega 3 PUFA (ω3) or PBS (fl ω3/ fl PBS group)

Colours red and green represent increment and decrement of protein abundance, respectively; with different colour intensities that correspond to the degree of differential expression. Proteins are annotated according to their cellular compartments and are depicted as different shapes, representing the functional classes of the proteins.

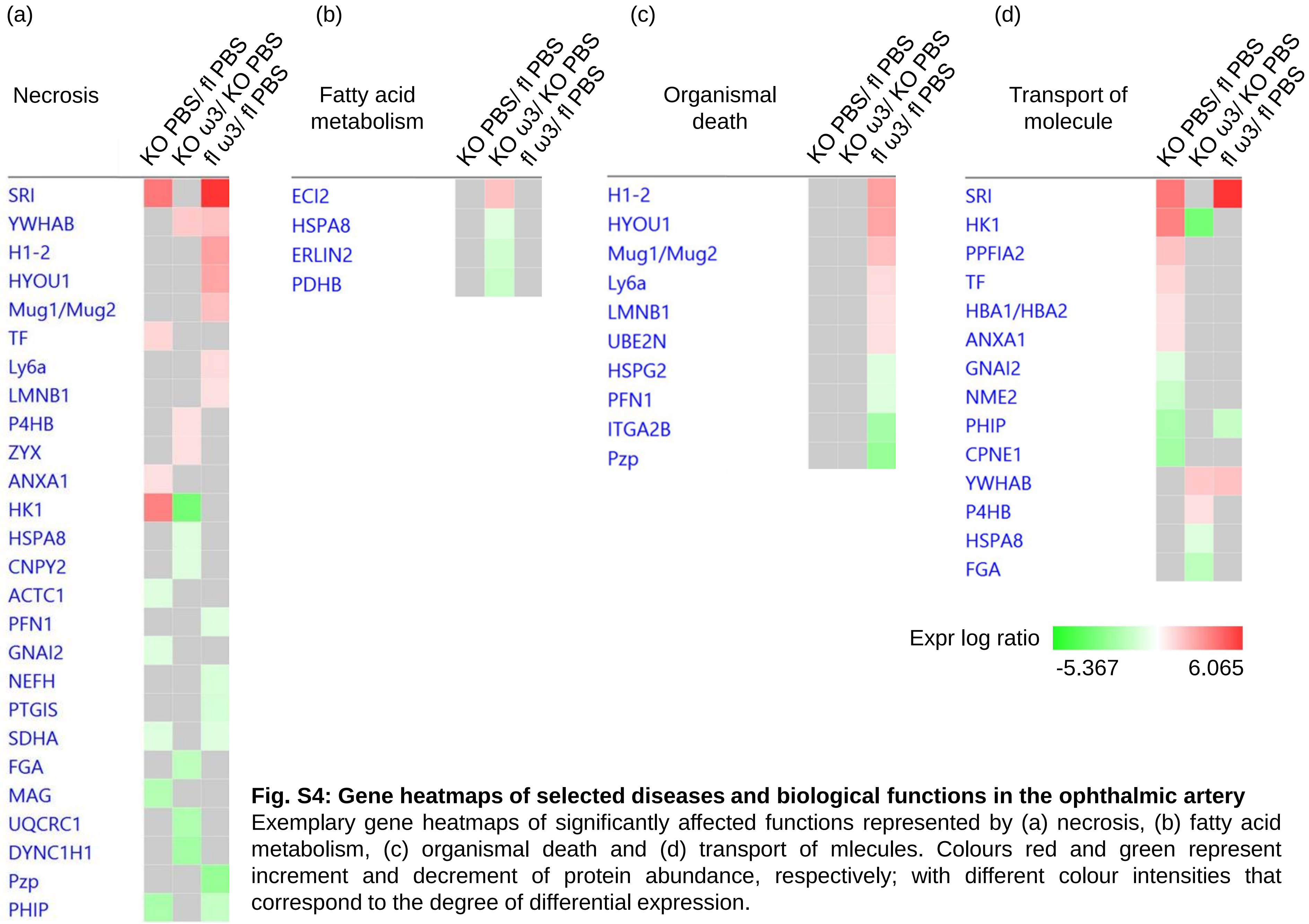

Fig. S4: Gene heatmaps of selected diseases and biological functions in the ophthalmic artery
Exemplary gene heatmaps of significantly affected functions represented by (a) necrosis, (b) fatty acid metabolism, (c) organismal death and (d) transport of mlecules. Colours red and green represent increment and decrement of protein abundance, respectively; with different colour intensities that correspond to the degree of differential expression.

(b)

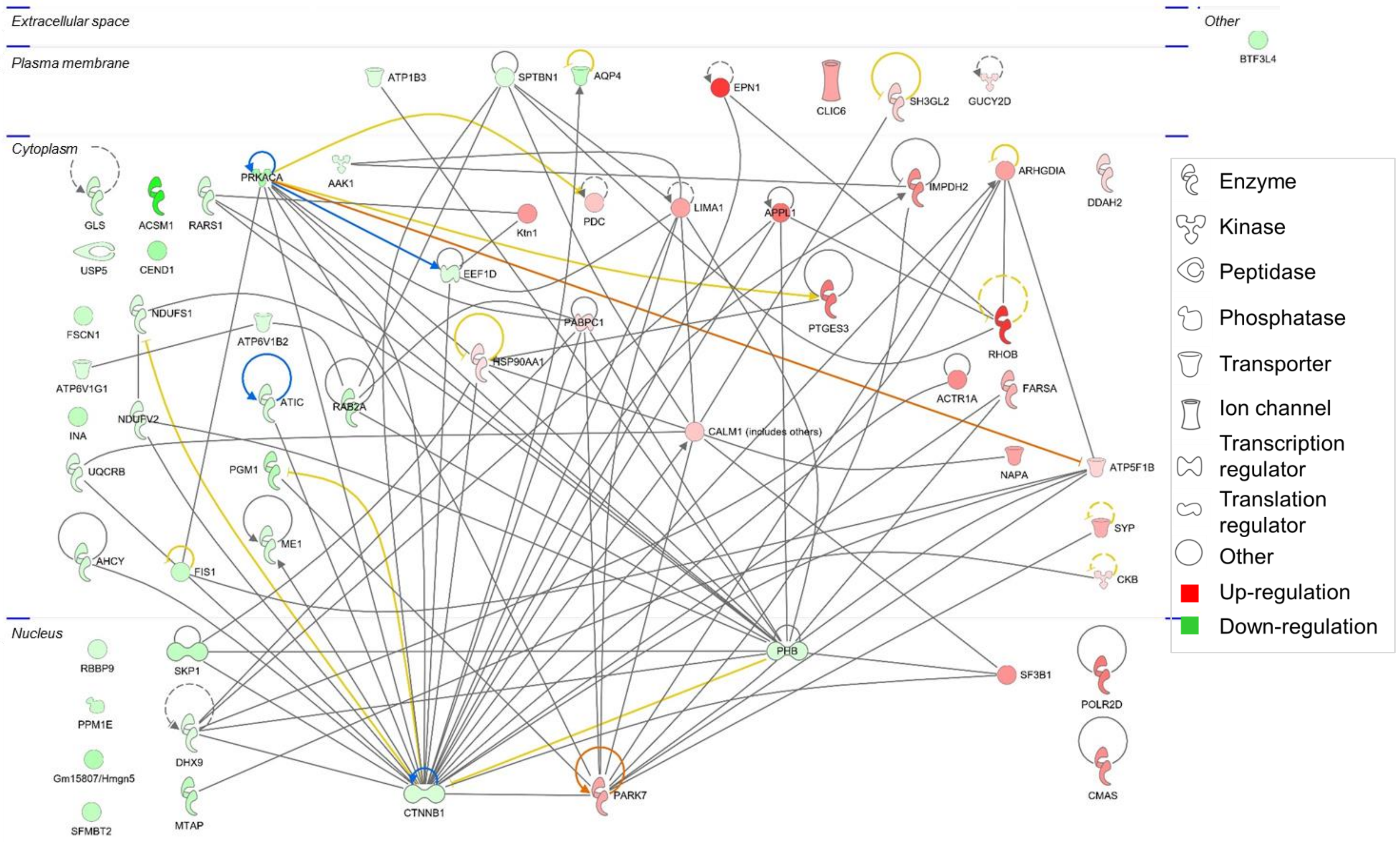

Fig. S5: Protein-protein interaction (PPI) network of the differentially expressed retinal proteins of the floxed mice following daily oral gavage for 7 days with either omega 3 PUFA (ω3) or PBS (fl ω3/ fl PBS)

Colours red and green represent increment and decrement of protein abundance, respectively; with different colour intensities that correspond to the degree of differential expression. Proteins are annotated according to their cellular compartments and are depicted as different shapes, representing the functional classes of the proteins.

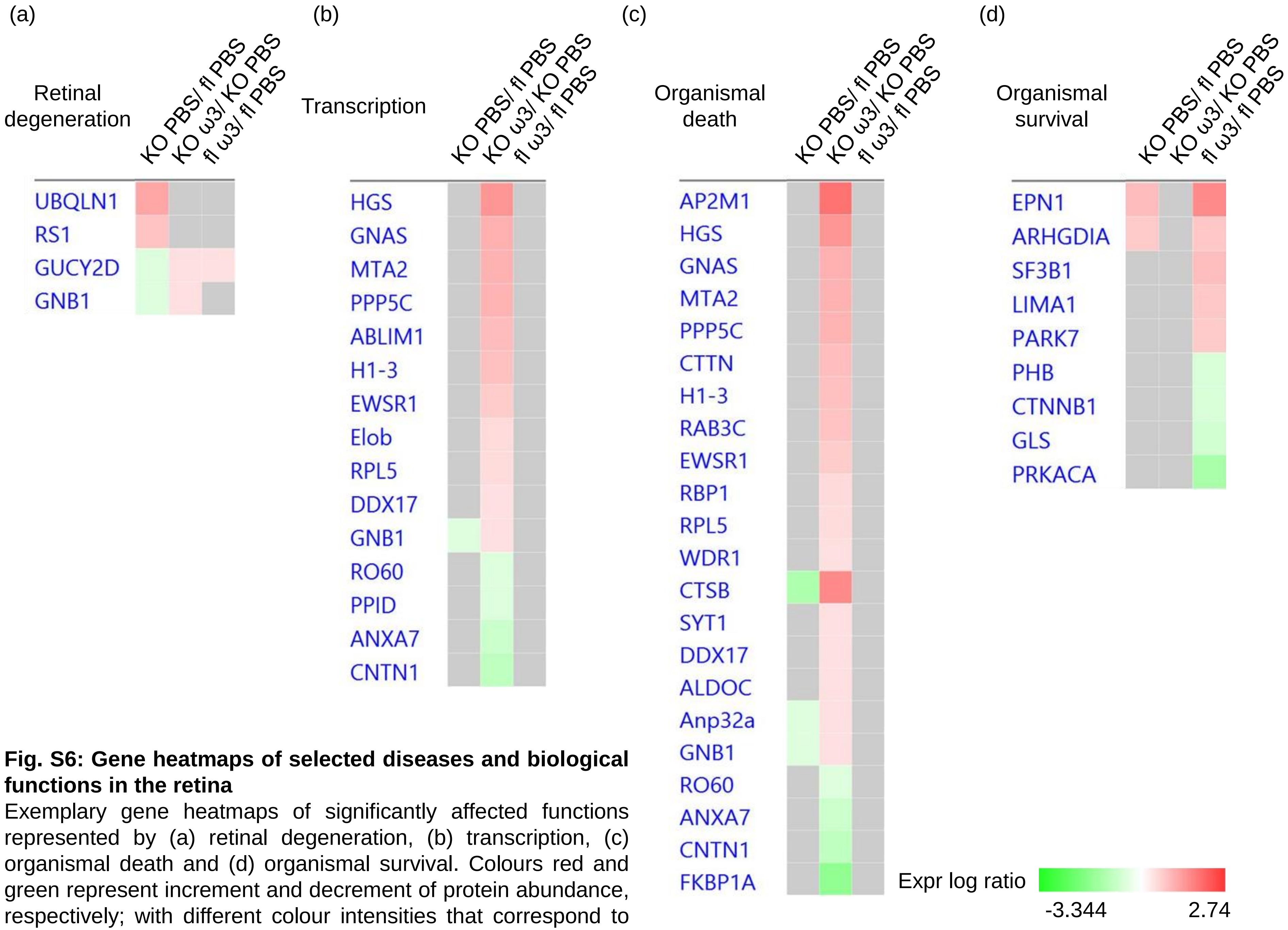

Fig. S6: Gene heatmaps of selected diseases and biological functions in the retina

Exemplary gene heatmaps of significantly affected functions represented by (a) retinal degeneration, (b) transcription, (c) organismal death and (d) organismal survival. Colours red and green represent increment and decrement of protein abundance, respectively; with different colour intensities that correspond to the degree of differential expression.
